# ATP-fueled STING activation of manganese coordinated nanoagonist to boost antitumor immunity

**DOI:** 10.1016/j.bioactmat.2026.02.012

**Published:** 2026-02-10

**Authors:** Si-Yao Han, Sui-Juan Zheng, Jia-Qi Luo, Hui-Han Yu, Xiao-Yue Liu, Jin-Zhi Du

**Affiliations:** a School of Medicine, South China University of Technology, Guangzhou, 510006, China; b School of Biomedical Sciences and Engineering, Guangzhou International Campus, South China University of Technology, Guangzhou, 511442, China; c National Engineering Research Center for Tissue Restoration and Reconstruction, and Guangdong Provincial Key Laboratory of Biomedical Engineering, South China University of Technology, Guangzhou, 510006, China; d Key Laboratory of Biomedical Materials and Engineering of the Ministry of Education, and Guangdong Provincial Key Laboratory of Biomedical Engineering, South China University of Technology, Guangzhou, 510006, China

**Keywords:** Adenosine triphosphate, Cancer metalloimmunotherapy, cGAS-STING activation, Coordination nanoparticles, Manganese ions

## Abstract

The cyclic GMP-AMP synthase-stimulator of interferon genes (cGAS-STING) pathway represents a central driver of innate immune activation, and manganese ion (Mn^2+^) has recently been identified as a potent modulator of this signaling axis. However, the application of Mn^2+^ is limited by its rapid clearance, nonspecific distribution and potential neurotoxicity. Inspired by the unique chemical structure and biological functions of adenosine triphosphate (ATP), we herein propose an ATP-Mn coordination nanoparticle (ATP-Mn CNP) to fuel cGAS-STING activation and antitumor immunity. We demonstrated that the phosphate groups of ATP could coordinate with Mn^2+^ and form stable, well-defined nanoparticles after lipid coating. ATP-Mn CNP significantly increased the expression of cGAS-STING-associated genes and activated the corresponding signaling cascades, and thus effectively polarized macrophages from tumor-supportive M2 to antitumor M1 phenotype. *In vivo* antitumor studies indicated that ATP-Mn CNP treatment significantly suppressed tumor growth, and reprogramed macrophages in tumors and draining lymph nodes, which thus facilitated the tumor infiltration of cytotoxic lymphocytes. Combination of ATP-Mn CNP with immune checkpoint inhibitors achieved 37.5% tumor eradication in MC38 murine models, and significantly prolonged mice survival. This study establishes an ATP-fueled coordination strategy that harnesses ATP as both an assembly ligand and an immune stimulator to enhance Mn-mediated STING activation.

## Highlights

• ATP-Mn coordination nanoparticle (ATP-Mn CNP) synergistically potentiates cGAS-STING activation for cancer immunotherapy.
• ATP not only drives the nanoparticle formation but synergizes with Mn^2+^ to amplify STING signaling.
• ATP-Mn CNP reprograms macrophages and enhances cytotoxic lymphocyte infiltration in tumors.
• Combining ATP-Mn CNP with checkpoint blockade achieves durable tumor suppression.

## Graphical abstract

**Figure:**
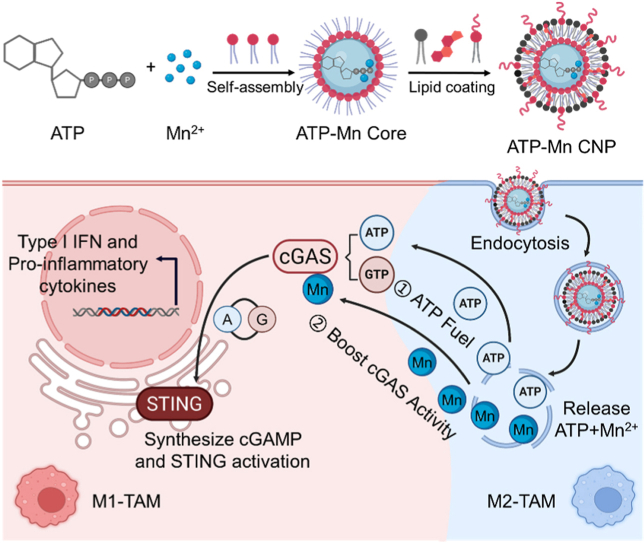

## 1 Introduction

Immunotherapy is revolutionizing cancer treatment [[1], [2], [3]], but still faces the challenge of low clinical response rates [[4], [5], [6]] due to the immunosuppressive tumor microenvironment and insufficient T cell infiltration [7,8]. Recent studies have shown that the cyclic GMP-AMP synthase-stimulator of interferon genes (cGAS-STING) pathway plays an important role in initiating anti-tumor immune responses and converting ‘cold’ tumors into ‘hot’ ones [[9], [10], [11]]. Manganese ion (Mn^2+^), an essential trace element involved in energy metabolism, bone formation, and neurotransmission [12,13], has recently emerged as a potent modulator of the STING pathway [14]. Mn^2+^ potentiates the cGAS-STING pathway by not only strengthening the binding affinity of cGAS to dsDNA but also directly promoting cGAS activation, leading to efficient catalysis of ATP and GTP into the second messenger cyclic GMP-AMP (cGAMP) and subsequent robust activation of STING signaling [[15], [16], [17]]. Consequently, Mn^2+^ has been recognized as an effective STING agonist with promising applications in cancer immunotherapy and vaccine adjuvants [18,19]. However, the application of free Mn^2+^ is significantly constrained by its rapid systemic clearance and nonspecific biodistribution, which typically results in inadequate tumor accumulation and potential neurotoxicity [[20], [21], [22]].

Various nanoscale delivery platforms have been developed by formulating Mn^2+^ into nanoparticles to enhance its tumor-targeted immunotherapy [[23], [24], [25]]. For example, a Mn-phenolic network was developed to achieve the dual functions of radiosensitization and STING pathway activation [26]. A hybrid mRNA vaccine was constructed by integrating small-sized manganese oxide with lipid nanoparticles, and the potency of the vaccine was significantly amplified by Mn^2+^-mediated STING activation [27]. Furthermore, the selection of an appropriate ligand can act synergistically with Mn^2+^ to augment the anti-tumor immune response. In a typical study, a coordination nanoparticle of c-di-AMP (CDA) with Mn^2+^ was developed, and the results showed that CDA-Mn^2+^ synergistically amplified the STING pathway to elicit robust anti-tumor immunity [28]. This study advocates the viability of elaborate ligands to promote Mn-mediated STING activation. Besides these, numerous nanodelivery platforms that combine Mn^2+^ with exogenous STING agonists have been reported [29,30]. It can't deny that the accessibility of STING agonists (e.g., CDA or MSA-2) is limited due to their high cost or complicated synthesis [14,31,32]. Consequently, it is urgently needed to identify readily available, cost-effective ligands to coordinate with Mn^2+^ to simultaneously boost its cGAS-STING activation.

Adenosine triphosphate (ATP), one of the most famous molecules in living organisms, not only functions as the universal energy currency for intracellular energy transfer but also serves as signaling mediators in diverse physiological processes [[33], [34], [35]]. In the context of cGAS-STING pathway, studies have revealed that ATP is an essential substrate for cGAS to synthesize the second messenger cGAMP [[36], [37], [38]]. Additionally, ATP also behaves as a key metabolic regulator to directly promote STING activation [39]. More interestingly, ATP has a unique chemical structure beyond its biological functions. Its multi-toothed phosphate groups provide excellent coordination sites with metal ions. Thus, ATP is an ideal ligand owing to its ready availability, high biocompatibility, multiple coordination sites, and its role as the natural substrate of cGAS. Several ATP-based coordination systems have been established. For instance, ATP-Cu^2+^ complexes have been employed to construct efficient catalytic systems, while ATP-Ce^3+^ coordination could form self-organized nanoparticles for ultrasensitive H_2_O_2_ and glucose detection [[40], [41], [42]]. However, the coordination of ATP with Mn^2+^ to activate the STING pathway for cancer immunotherapy remains unexplored.

**Fig. 1:**
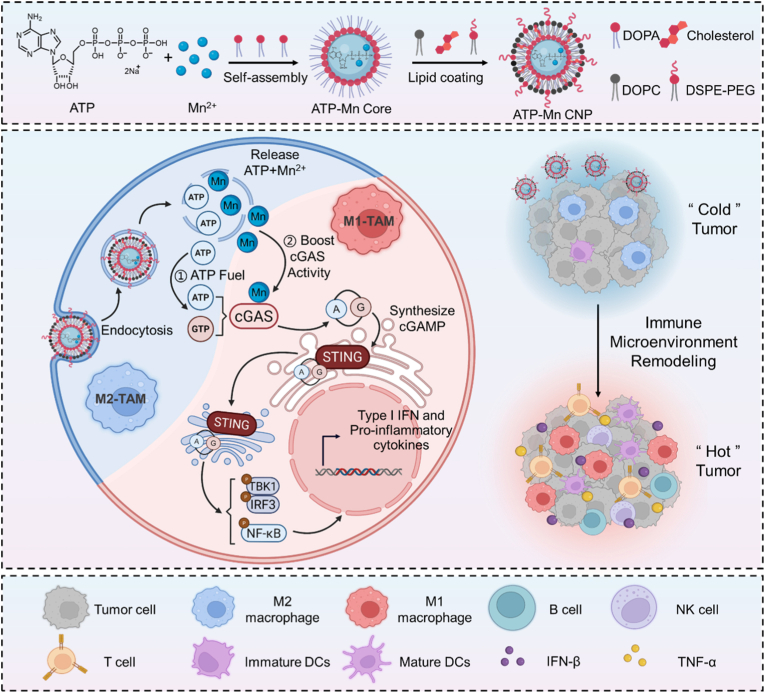
**Schematic illustration of the preparation of ATP-Mn CNP and its immunostimulatory mechanism.** Mn^2+^, ATP, and 1,2-dioleoyl-sn-glycero-3-phosphate (DOPA) spontaneously self-assemble into a hydrophobic core nanoparticle, which is subsequently encapsulated by 1,2-dioleoyl-sn-glycero-3-phosphocholine (DOPC), cholesterol, and 1,2-diastearoyl-sn-glycero-3-phosphoethanolamine-N-[amino (polyethylene glycol)2000] (DSPE-PEG2000) to generate ATP-Mn CNP. Following cellular uptake *via* endocytosis, the acidic endosomal environment triggers rapid cytosolic release of ATP and Mn^2+^, which synergistically activates the cGAS-STING signaling pathway, leading to M2-to-M1 macrophage repolarization as well as enhanced secretion of type I interferons and proinflammatory cytokines. This cascade ultimately promotes the infiltration of tumor-killing immune cells into tumor tissues, and achieves efficient cancer immunotherapy (Created with Biorender.com).

Herein, we hypothesize that ATP can interact with Mn^2+^ to form a coordinated nanoagonist. To construct such a system, ATP and Mn^2+^ were first co-assembled into a core nanoparticle *via* a reverse microemulsion process, followed by stabilization with lipid coating, yielding ATP-Mn coordination nanoparticles (ATP-Mn CNP). Unlike conventional inorganic phosphate ligands, ATP not only drives the nanoparticle formation but synergizes with Mn^2+^ to amplify cGAS-STING signaling (Fig. 1). Cellular studies justify that ATP-Mn CNP demonstrates stronger STING signaling activation, enhanced dendritic cell maturation and macrophage repolarization, as well as the secretion of type I interferons and pro-inflammatory cytokines in comparison with free Mn^2+^ and tripolyphosphate (TPP)-Mn CNP controls. Intravenous administration of ATP-Mn CNP elicited potent antitumor immunity in B16F10 and MC38 tumors. Combination therapy with anti-PD-1 achieved 37.5% tumor eradication, and achieved durable systemic immune response in MC38 tumor models. Our study proposes an ATP-fueled bio-coordination strategy that harnesses ATP as both an energetic and structural ligand to drive Mn-based STING activation.

## 2 Results and discussion

### 2.1 Preparation, characterization and cellular uptake of ATP-Mn CNP

**Fig. 2:**
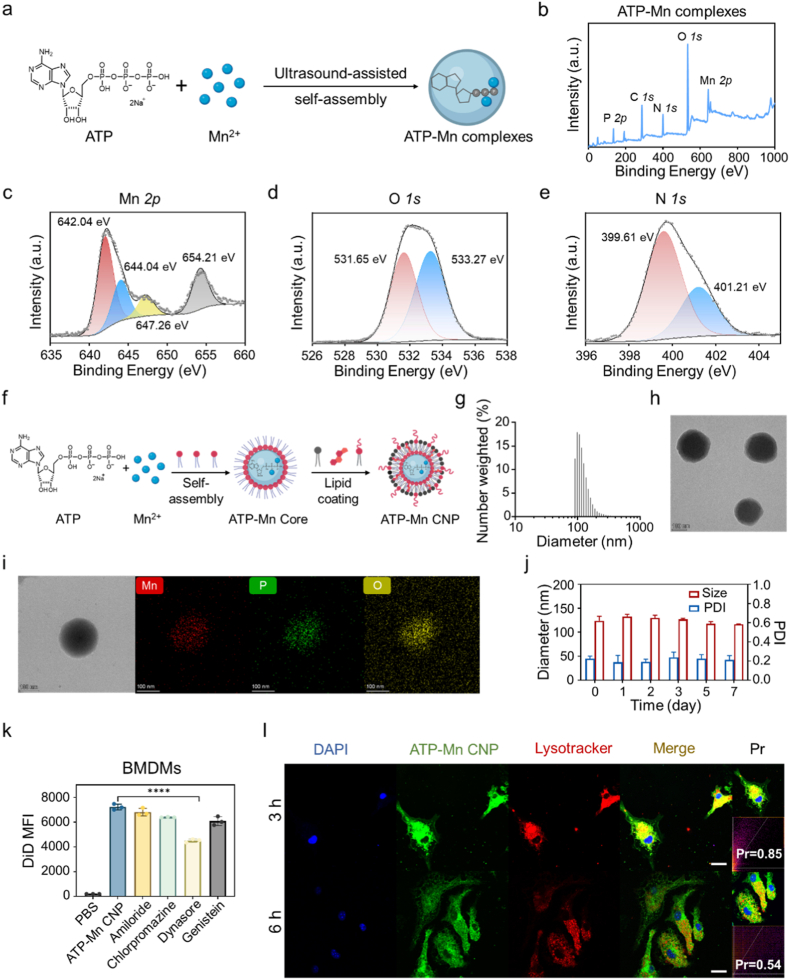
**Preparation, characterization and cellular uptake of ATP-Mn CNP.** (a) Schematic illustration of the preparation of ATP-Mn complexes. (b) Full X-ray photoelectron spectroscopy (XPS) spectrum of ATP-Mn complexes. High-resolution XPS spectra of (c) Mn *2p*, (d) O *1s*, and (e) N *1s* of ATP-Mn complexes. Arbitrary units (a. u.). (f) Schematic illustration of the preparation of ATP-Mn CNP. (g) Size distribution of ATP-Mn CNP determined by dynamic light scattering (DLS) measurement. (h) Transmission electron microscopy (TEM) image of ATP-Mn CNP. Scale bar = 100 nm. (i) Energy dispersive X-ray spectroscopy of ATP-Mn CNP. Scale bar = 100 nm. (j) Stability evaluation of ATP-Mn CNP in 10% FBS over 7 days (n = 3). (k) Cellular uptake of ATP-Mn CNP by BMDMs after pre-treatment with different endocytosis inhibitors (n = 3). (l) Cellular uptake and lysosomal colocalization of BMDMs after incubation with ATP-Mn CNP for 3 or 6 h. Nuclei were stained with DAPI (blue), DiD-labeled ATP-Mn CNP were presented as green, and lysosomes were stained with LysoTracker (red). Scale bar = 20 μm. Data are presented as mean ± SD. ∗∗∗∗P < 0.0001.

The phosphate groups of ATP are thought to coordinate with Mn^2+^. To demonstrate this, we simply mixed the aqueous solutions of ATP and Mn^2+^ together under ultrasound and obtained white precipitate after centrifugation (Fig. 2a). The sample was subjected to X-ray photoelectron spectroscopy (XPS) measurement with ATP and MnCl_2_·4H_2_O as controls. In the spectra, Mn and O signals were detected in MnCl_2_·4H_2_O, while ATP exhibited characteristic peaks corresponding to the C, N, O, and P elements (Fig. S1). The coexistence of C, N, O, P, and Mn signals in the ATP-Mn complexes confirmed the successful incorporation of Mn^2+^ into the ATP framework (Fig. 2b). High-resolution analysis of the Mn *2p* region revealed that the Mn *2p* peak shifted from 641.60 eV in MnCl_2_·4H_2_O to 642.04 eV in ATP-Mn complexes, accompanied by the appearance of a higher-binding-energy component (from 643.28 eV to 644.04 eV) and similar satellite structures (Fig. 2c and S2). These features indicate a pronounced alteration in the local electronic environment of Mn upon coordination with ATP. In the O *1s* spectra, ATP displayed two characteristic peaks at 531.04 and 532.87 eV. After coordination with Mn^2+^ to form ATP-Mn complexes, the peaks shifted to higher binding energies at 531.65 and 533.27 eV, respectively (Fig. 2d and S2). Such a shift toward higher binding energy indicates the decrease in electron density around the oxygen atoms due to coordination with Mn^2+^, confirming the formation of Mn-O bonds between Mn^2+^ and the phosphate groups of ATP. Meanwhile, the N *1s* spectra of ATP and ATP-Mn complexes showed negligible changes, indicating that the nitrogen atoms of adenine were not significantly involved in coordination (Fig. 2e and S2). Collectively, these results demonstrate that Mn^2+^ can coordinate with the phosphate oxygen atoms of ATP.

However, direct mixing of ATP and Mn^2+^ did not form uniform particles. The ATP-Mn complexes prepared *via* ultrasonication exhibited large particle sizes (Fig. S3). To make uniform and stable ATP-Mn coordination nanoparticles (ATP-Mn CNP), a reverse microemulsion approach was employed, and lipid molecules were introduced through a two-step procedure (Fig. 2f). Initially, the phosphate groups of ATP were cross-linked with Mn^2+^ ions in a reverse microemulsion containing Triton X-100/hexanol/cyclohexane in the presence of 1,2-dioleoyl-sn-glycero-3-phosphate (DOPA). The polar lipid DOPA capped the particles, yielding the ATP-Mn core with a hydrophobic surface. Subsequently, the ATP-Mn core was coated with a lipid mixture of 1,2-dioleoyl-sn-glycero-3-phosphocholine (DOPC), cholesterol, and 1,2-diastearoyl-sn-glycero-3-phospho-ethanolamine-N-[amino (polyethylene glycol)2000] (DSPE-PEG2000) *via* hydrophobic interactions to form ATP-Mn CNP. After lipid coating, ATP-Mn CNP became water soluble and was used for the following biological studies. Inductively coupled plasma mass spectrometry (ICP-MS) analysis revealed an approximately 1:1 molar ratio of manganese to phosphorus in the core, and the mass fraction of Mn in ATP-Mn CNP was calculated to be 14.18%. Characterization by dynamic light scattering (DLS) and transmission electron microscopy (TEM) revealed that ATP-Mn CNP possessed a uniform spherical morphology with a core-shell architecture and a diameter of approximately 110 nm (Fig. 2g and h). The surface charge, as determined by zeta potential measurements, was approximately −10 mV (Fig. S4). The energy dispersive X-ray spectroscopy (EDS) showed the homogeneous distributions of Mn ions within ATP-Mn CNP (Fig. 2i). In addition, stability assessments in 10% fetal bovine serum (FBS) solution indicated favorable colloidal stability, with negligible changes in particle size and polydispersity index (PDI) over seven days (Fig. 2j).

Next, DiD-labeled ATP-Mn CNP was prepared to study the cellular uptake in bone-marrow-derived macrophages (BMDMs) and bone-marrow-derived dendritic cells (BMDCs). Flow cytometry analysis revealed time-dependent internalization of DiD-labeled ATP-Mn CNP in both BMDMs and BMDCs (Fig. S5). Mechanistic studies indicated that ATP-Mn CNP uptake predominantly occurred *via* dynamin-dependent pathways, including clathrin-mediated and endophilin-assisted endocytosis (Fig. 2k and S6). Since the activation of the STING pathway occurs within the cytosol, efficient endosomal escape of the nanoparticles is essential for their immunostimulatory function. Many phosphate-coordinated nanoparticles show pH-responsive degradability and drug-release behavior, which are important for biomedical applications [[43], [44], [45]]. To assess the acidic pH-sensitivity of ATP-Mn CNP, we compared the release kinetics of Mn^2+^ at pH 7.4 (simulated normal physiological pH) and 5.7 (simulated endosomal pH) (Fig. S7). This result indicated that Mn^2+^ can be released under the stimulation of acidic endosomal pH, which may be beneficial for its biological functions. TEM imaging was also performed at neutral (pH 7.4) and acidic (pH 5.7) pHs to evaluate the structural integrity of ATP-Mn CNP (Fig. S8). As indicated, ATP-Mn CNP disintegrated into irregular fragments after incubation at endosomal pH. This acid-responsive behavior is supposed to facilitate cytoplasmic delivery of ATP and Mn^2+^. Confocal microscopy showed that the nanoparticles initially colocalized with acidic endosomes. Over time, the particle gradually dispersed into cytoplasm with Pearson's correlation coefficient (Pr) decreasing from 0.85 to 0.54, indicating efficient endosomal escape of ATP-Mn CNP (Fig. 2l). Cytotoxicity assays on BMDMs and BMDCs and hemolysis tests confirmed good biocompatibility of these particles (Fig. S9).

### 2.2 ATP-Mn CNP promotes M1 macrophage polarization and BMDCs activation

**Fig. 3:**
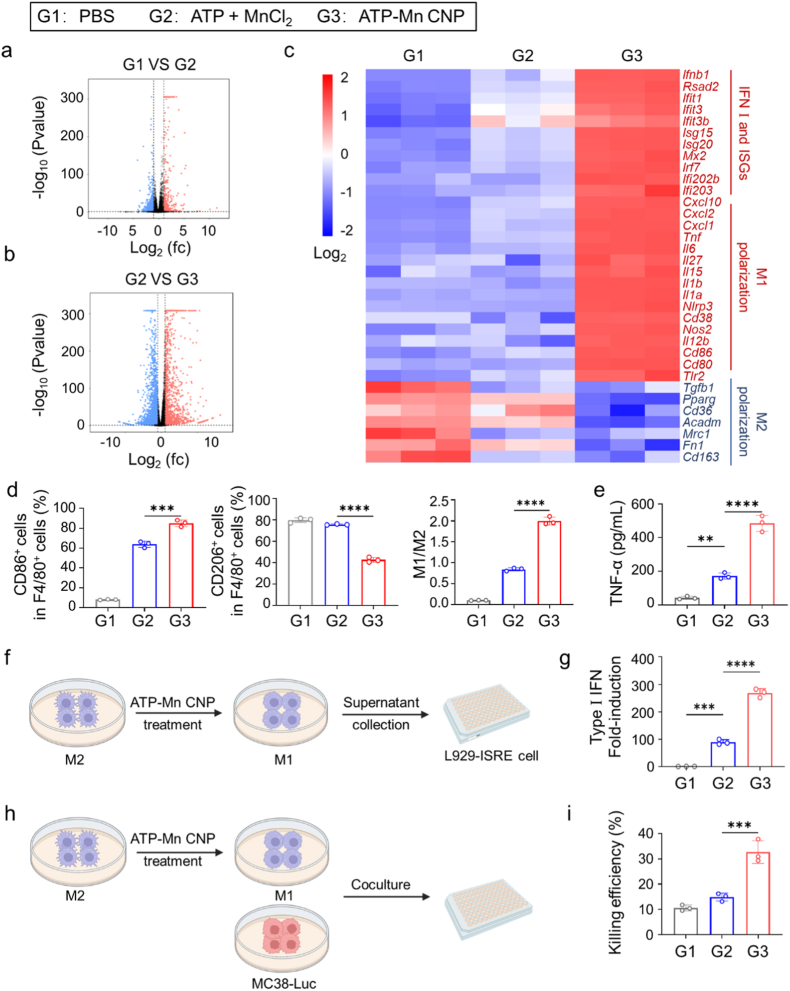
***In vitro* analysis of macrophage activation and functional responses induced by ATP-Mn CNP treatment.** (a-b) Differentially expressed genes (DEGs) identified by mRNA-seq analysis (q < 0.05) between ATP + MnCl_2_ and PBS, and between ATP + MnCl_2_ and ATP-Mn CNP (n = 3). (c) Summary of DEGs related to the cGAS-STING pathway in response to ATP-Mn CNP treatment, including type I interferons (IFN), interferon-stimulated genes (ISGs), M1 polarization markers, and M2 polarization markers. (d) Flow cytometry analysis shows the percentages of M1 (CD86^+^) and M2 (CD206^+^) macrophages in BMDMs treated with PBS, ATP + MnCl_2_, or ATP-Mn CNP for 24 h (n = 3). (e) TNF-α levels in culture supernatants of BMDMs, measured by ELISA (n = 3). (f) Schematic illustration of the type I IFN assay. (g) Type I IFN activity in culture supernatants from BMDMs treated with PBS, ATP + MnCl_2_, or ATP-Mn CNP for 24 h. (h) Schematic illustration of the tumor cell killing assay. (i) Cytotoxic activity of BMDMs toward MC38-Luc cells after the indicated treatments. BMDMs were first treated with PBS, ATP + MnCl_2_, or ATP-Mn CNP for 24 h, followed by co-culture with MC38-Luc cells for another 24 h. Luciferase activity was then measured to quantify tumor cell killing (n = 3). Data are shown as the mean ± SD. ∗P < 0.05, ∗∗P < 0.01, ∗∗∗P < 0.001, and ∗∗∗∗P < 0.0001.

To elucidate the synergistic interaction between ATP and Mn^2+^, we first evaluated the individual and combined effects of these two components on BMDCs and BMDMs at concentrations matching those in ATP-Mn CNPs. ATP or Mn^2+^ alone elicited only weak cellular activation, whereas their combination markedly enhanced the activation of both cell types and potently stimulated type I interferon (IFN) secretion (Fig. S10). Subsequently, we conducted functional investigations on ATP-Mn CNP. First, to elucidate the immunostimulatory mechanisms of ATP-Mn CNP, transcriptomic profiling was performed on BMDMs treated with ATP-Mn CNP, free ATP + MnCl_2_, or PBS. RNA-sequencing analysis revealed distinct transcriptional responses among these treatment groups. Compared with PBS, treatment with ATP + MnCl_2_ upregulated 601 genes and downregulated 344 genes (Fig. 3a). In contrast, ATP-Mn CNP treatment induced more extensive transcriptomic changes, with 2759 genes upregulated and 1470 genes downregulated relative to the ATP + MnCl_2_ group (Fig. 3b). Differential expression analysis indicated marked activation of genes involved in the cGAS-STING signaling pathway and M1 macrophage polarization, accompanied by the suppression of M2-associated genes (Fig. 3c). Gene set enrichment analysis (GSEA) further confirmed significant enrichment of M1 macrophage polarization and the type I IFN response in the ATP-Mn CNP group relative to the ATP + MnCl_2_ group (Fig. S11). These findings suggest that ATP-Mn CNP not only enhances cGAS-STING pathway activation but also promotes a shift toward proinflammatory M1 macrophage polarization.

Flow cytometry analysis was employed to verify the immunomodulatory effects of ATP-Mn CNP in BMDMs and BMDCs. As indicated, ATP-Mn CNP treatment significantly upregulated the costimulatory molecule CD86 while downregulating the M2 marker CD206 of BMDMs. Compared with free ATP + MnCl_2_, ATP-Mn CNP increased the M1/M2 ratio by approximately 3-fold (Fig. 3d and S12). This is consistent with previous studies showing that the cGAS-STING signaling pathway is one of the critical pathways for macrophage repolarization [46,47]. Furthermore, reverse transcription quantitative real-time PCR (RT-qPCR) analysis of polarization-related genes in BMDMs revealed that ATP-Mn CNP markedly downregulated *Arg1* expression and upregulated *Nos2* and *Cxcl10* expression, thereby confirming its ability to repolarize macrophages (Fig. S13). For BMDCs, ATP-Mn CNP enhanced the expression of activation markers such as CD80 and CD86, and increased the proportion of CD80^+^CD86^+^ cells within CD11c^+^ populations (Fig. S14). Supernatants from ATP-Mn CNP-treated BMDMs were subsequently analyzed by enzyme-linked immunosorbent assay (ELISA). The secretion of tumor necrosis factor (TNF)-α was significantly elevated in both ATP-Mn CNP and ATP + MnCl_2_ groups (Fig. 3e). ATP-Mn CNP induced approximately 2-fold higher TNF-α levels than ATP + MnCl_2_ at the same Mn concentration. This indicated that ATP-Mn CNP significantly enhanced the capacity of BMDMs to secrete pro-inflammatory cytokines.

Given that the production of type I IFN serves as a key downstream hallmark of cGAS-STING pathway activation [46,48], we next evaluated the ability of ATP-Mn CNP to induce IFN secretion using an L929 interferon-stimulated response element (ISRE) reporter system. The assay utilized L929 cells stably transfected with ISRE driving luciferase expression. Conditioned media from BMDCs or BMDMs treated with ATP + MnCl_2_ or ATP-Mn CNP were collected and incubated with the reporter cells for 4 h before luciferase quantification (Fig. 3f). In BMDMs, ATP-Mn CNP markedly enhanced type I IFN secretion, reaching approximately 3-fold higher levels than those of ATP + MnCl_2_ group (Fig. 3g). In contrast, both ATP + MnCl_2_ and ATP-Mn CNP elicited robust secretion of type I IFN in BMDCs (Fig. S15). This observation was consistent with flow cytometry data, which demonstrated enhanced activation and more pronounced M1 repolarization of BMDMs. The macrophage-specific enhancement was likely due to the higher phagocytic tendency of macrophages for particles. We next examined whether ATP-Mn CNP could enhance macrophage-mediated tumor cell killing (Fig. 3h). BMDMs pretreated with various formulations were co-cultured with B16-Luc or MC38-Luc tumor cells, and the cytotoxicity was assessed *via* luciferase activity measurement. ATP-Mn CNP treatment significantly enhanced the cytotoxic activity of BMDMs against both tumor cell lines compared with ATP + MnCl_2_ (Fig. 3i and S16). Furthermore, ATP-Mn CNP promoted the phagocytosis of B16 tumor cells by macrophages, increasing the phagocytosis rate of B16 cells by BMDMs from 8% to 20% (Fig. S17). Collectively, these results indicate that ATP-Mn CNP enhances macrophage antitumor activity by promoting type I IFN and TNF-α secretion and facilitating tumor cell phagocytosis. Overall, ATP-Mn CNP outperforms free ATP + MnCl_2_ in activating macrophages at equivalent Mn and ATP concentrations.

### 2.3 ATP amplifies the activation of cGAS-STING pathway and immunostimulation

**Fig. 4:**
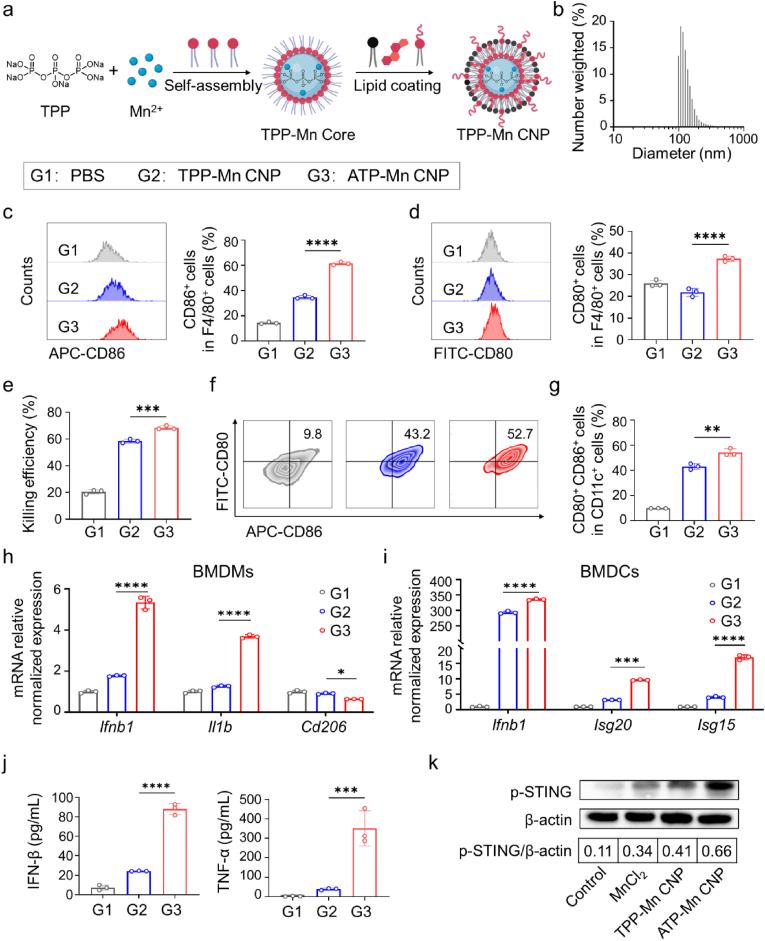
***In vitro* evaluation of immune cell activation and STING pathway responses induced by ATP-Mn CNP and TPP-Mn CNP.** (a) Schematic illustration of the synthesis of TPP-Mn CNP. (b) Size distribution of TPP-Mn CNP determined by DLS. (c, d) Flow cytometry analysis and quantification of activated BMDMs (CD80^+^, CD86^+^) after 24 h incubation with PBS, TPP-Mn CNP, or ATP-Mn CNP at indicated concentrations (n = 3). (e) Killing efficiency of MC38-Luc cells co-cultured with BMDMs pre-treated with PBS, TPP-Mn CNP, or ATP-Mn CNP (n = 3). (f, g) Flow cytometry analysis and quantification of matured BMDCs (CD80^+^CD86^+^) after 24 h treatment with PBS, TPP-Mn CNP, or ATP-Mn CNP (n = 3). (h) Relative mRNA expression levels of *Ifnb1*, *Il1b*, and *Cd206* in BMDMs after 12 h treatment with PBS, TPP-Mn CNP, or ATP-Mn CNP, measured by RT-qPCR (n = 3). (i) Relative mRNA expression levels of *Ifnb1*, *Isg20*, and *Isg15* in BMDCs after 12 h treatment with PBS, TPP-Mn CNP, or ATP-Mn CNP, measured by RT-qPCR (n = 3). (j) Concentrations of IFN-β and TNF-α in BMDM culture supernatants determined by ELISA after 24 h treatment (n = 3). (k) Western blot analysis of the expression and phosphorylation of STING protein in BMDMs after 12 h treatment with PBS, MnCl_2_, TPP-Mn CNP, or ATP-Mn CNP (n = 3). Data are shown as the mean ± SD. ∗P < 0.05, ∗∗P < 0.01, ∗∗∗P < 0.001, and ∗∗∗∗P < 0.0001.

To clarify the role of ATP in cGAS-STING pathway activation, we replaced ATP with biologically inert sodium tripolyphosphate (TPP) as the ligand of Mn^2+^ to prepare control nanoparticles (TPP-Mn CNP) following the same protocol as ATP-Mn CNP (Fig. 4a). Physicochemical characterization confirmed that TPP-Mn CNP and ATP-Mn CNP exhibited similar size distributions, surface charges, and morphologies (Fig. 4b and S18). Their influence on the activation or maturation of antigen-presenting cells (APCs) was compared. Although ICP-MS measurement indicated that BMDMs treated with ATP-Mn CNP showed even lower intracellular manganese level compared with the TPP-Mn CNP group (Fig. S19), ATP-Mn CNP significantly increased the proportion of M1-polarized cells, as indicated by elevated CD80^+^ and CD86^+^ populations relative to TPP-Mn CNP treatment (Fig. 4c and d), and concomitantly enhanced macrophage-mediated tumor cell killing (Fig. 4e). Similarly, trends were observed in BMDCs, where ATP-Mn CNP treatment induced higher activation and maturation of BMDCs than TPP-Mn CNP (Fig. 4f and g).

In addition, genes (*Ifnb1*, *Isg20*, *Isg15*) that closely correlate with cGAS-STING pathway [49,50] as well as the gene (*Cd206*) that closely correlates with M2 macrophage phenotype [51] at the mRNA level were measured by RT-qPCR. In BMDMs, treatment with ATP-Mn CNP increased *Ifnb1* and *Il1b* expression by approximately 3-fold compared with TPP-Mn CNP at equivalent Mn concentrations, while concomitantly reducing *Cd206* expression by approximately 40% (Fig. 4h). Similarly, ATP-Mn CNP enhanced the expression of *Ifnb*1, *Isg15*, and *Isg20* relative to TPP-Mn CNP in BMDCs (Fig. 4i). We next examined the differential secretion of type I IFN and proinflammatory cytokines in BMDMs following treatment with ATP-Mn CNP or TPP-Mn CNP. Cell culture supernatants were collected after 24 h and analyzed by ELISA (Fig. 4j). The results demonstrated that ATP-Mn CNP significantly enhanced IFN-β and TNF-α secretion compared with TPP-Mn CNP. To further investigate the activation of the STING signaling pathway, we analyzed the phosphorylation levels of STING, TBK1, and IRF3 proteins in BMDMs by Western blot (Fig. 4k and S20). Treatment with MnCl_2_ alone or TPP-Mn CNP moderately increased the phosphorylation of STING, TBK1, and IRF3. In contrast, ATP-Mn CNP induced more pronounced enhancement of phosphorylation of STING, TBK1, and IRF3. These results indicate that ATP incorporation significantly potentiates Mn-mediated cGAS-STING signaling pathway activation, consistent with the observed increases in type I IFN and proinflammatory cytokine secretion.

### 2.4 ATP-Mn CNP enhances antitumor efficacy in B16F10 melanoma model

**Fig. 5:**
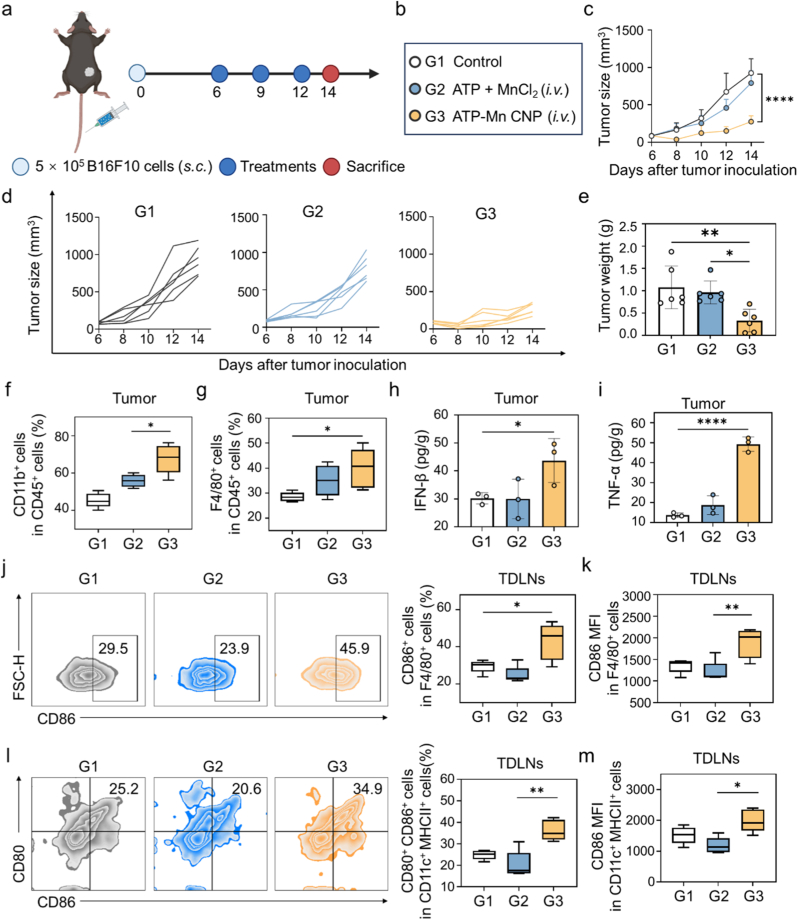
***In vivo* antitumor immunotherapy in** B16F10 **tumors.** (a-b) Schematic illustration of the treatment procedure. (c) Tumor growth curves of B16F10 tumor-bearing mice in different treatment groups. (d) Spider plots showing individual tumor growth curves for each mouse across the treatment groups. (e) Quantification of tumor weights in mice treated with PBS or various treatments (n = 6). (f-g) Flow cytometry analysis of the percentages of CD11b^+^ cells (f) and F4/80^+^ macrophages (g) within tumors (n = 5). (h-i) ELISA quantification of TNF-α (h) and IFN-β (i) levels in the tumor tissues (n = 3). (j) Percentage of CD86^+^ macrophages in tumor-draining lymph nodes after different treatments (n = 5). (k) Mean fluorescence intensity (MFI) of CD86 on macrophages in tumor-draining lymph nodes, quantified by flow cytometry (n = 5). (l) Percentage of CD80^+^ CD86^+^ DCs in tumor-draining lymph nodes after different treatments (n = 5). (m) CD80 MFI of DCs in tumor-draining lymph nodes (n = 5). Data are presented as the mean ± SD. Statistical significance is indicated as ∗P < 0.05, ∗∗P < 0.01, ∗∗∗P < 0.001, and ∗∗∗∗P < 0.0001.

B16F10 melanoma is recognized as a poorly immunogenic tumor type [52,53]. We established the B16F10 subcutaneous tumor model in C57BL/6 mice to evaluate the antitumor immune response of ATP-Mn CNP. When tumor volume reached approximately 100 mm^3^, mice were randomly divided into three groups: PBS control (G1), ATP + MnCl_2_ (2 mg/kg Mn, *i.v.*, G2), and ATP-Mn CNP (2 mg/kg Mn, *i.v.*, G3) (Fig. 5a and b). Treatments were administered every three days for a total of three injections. Tumor growth inhibition rates relative to the PBS group were calculated as 14.3% for G2 and 70.2% for G3, demonstrating a markedly enhanced therapeutic effect of ATP-Mn CNP over the free ATP + MnCl_2_ formulation (Fig. 5c). This trend was further validated by individual tumor trajectories and average tumor weights (Fig. 5d and e). To investigate the immunological changes induced by these treatments, we performed flow cytometry analysis of tumor-infiltrating immune cells on day 14 post-initial injection. As indicated, ATP-Mn CNP treatment significantly increased the proportion of CD11b^+^ myeloid cells and F4/80^+^ macrophages within the tumor tissues compared with both PBS and ATP + MnCl_2_ groups (Fig. 5f–g and S21). Concomitantly, the proportion of immunosuppressive regulatory T cells (Tregs) was reduced, suggesting a shift of the tumor microenvironment from immunologically “cold” to a more inflamed phenotype (Fig. S22). We next assessed tumor cytokine production by ELISA. ATP-Mn CNP induced markedly higher secretion of proinflammatory cytokines such as TNF-α and IFN-β in comparison with PBS and ATP + MnCl_2_ treatments (Fig. 5h and i), which is consistent with the enhanced activation of innate immune cells. Analysis of tumor-draining lymph nodes (TDLNs) revealed that ATP-Mn CNP treatment promoted macrophage repolarization toward the M1 phenotype and facilitated dendritic cell maturation, as indicated by elevated CD80^+^ and CD86^+^ populations (Fig. 5j–m and S23). Systemic immune activation was further corroborated by splenic immune profiling, which showed a significant increase in CD8^+^ cytotoxic T cells following ATP-Mn CNP administration (Fig. S24). These results demonstrate that ATP-Mn CNP not only exerts potent direct antitumor effects but also remodels the immunosuppressive tumor microenvironment, enhances systemic antitumor immunity, and significantly improve therapeutic efficacy compared with free ATP + MnCl_2_ treatment in the B16F10 melanoma model.

### 2.5 ATP-Mn CNP enhances antitumor efficacy in MC38 colorectal cancer model

**Fig. 6:**
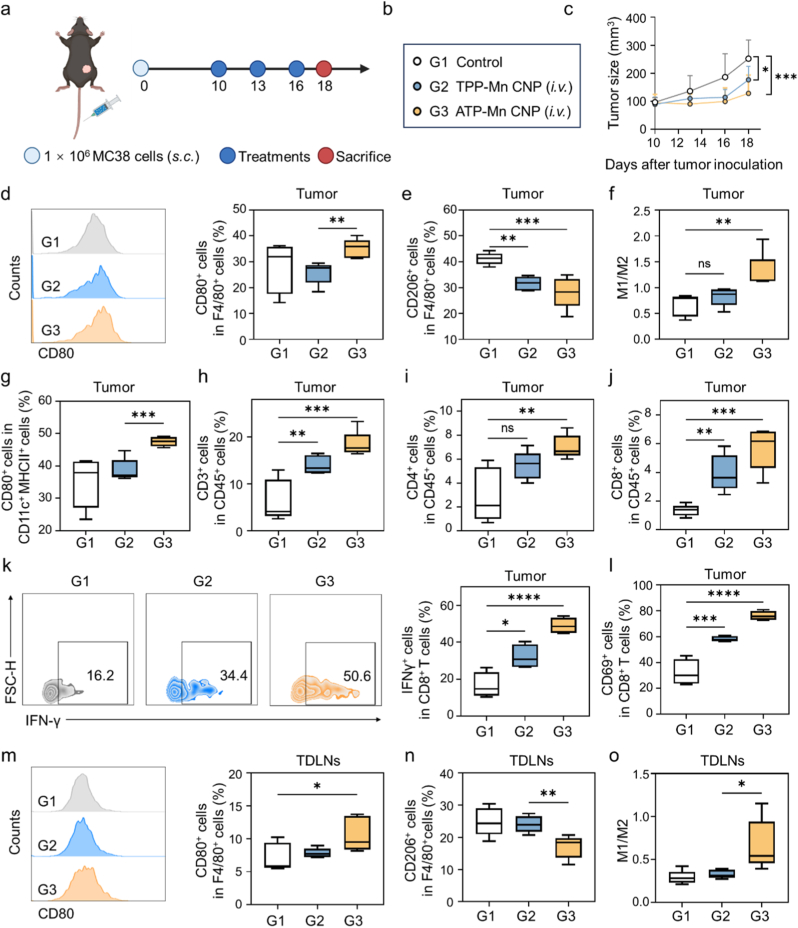
***In vivo* antitumor immunotherapy in MC38 model.** (a) Schematic illustration of the treatment process. (b-c) Tumor size curves of MC38 tumor-bearing mice in various treatment groups. (d) Percentages of CD80^+^ macrophages in tumor immune microenvironment (n = 5). (e) Percentages of CD206^+^ macrophages in tumor immune microenvironment (n = 5). (f) Ratio of M1 macrophages to M2 macrophages (n = 5). (g) Percentages of CD80^+^ DCs in tumor immune microenvironment (n = 5). (h-j) Percentages of CD3^+^ T cells (h), CD4^+^ T cells (i) and CD8^+^ T cells (j) in tumor immune microenvironment after different treatments (n = 5). (k-l) Percentages of CD80^+^ (k) and CD206^+^ (l) macrophages in TDLNs (n = 5). (m) Ratio of M1 macrophages to M2 macrophages in TDLNs (n = 5). Data are presented as the mean ± SD. Statistical significance is indicated as ∗P < 0.05, ∗∗P < 0.01, ∗∗∗P < 0.001, and ∗∗∗∗P < 0.0001.

To further evaluate the antitumor immune efficacy of ATP-Mn CNP, we employed a subcutaneous MC38 tumor model in C57BL/6 mice. When the tumor volume reached approximately 100 mm^3^, mice were randomly assigned to different treatment groups and received intravenous injections every three days for a total of three doses (Fig. 6a and b). The treatment regimens were defined as follows: G1: PBS; G2: TPP-Mn CNP (Mn dose: 2 mg/kg); and G3: ATP-Mn CNP (Mn dose: 2 mg/kg). As indicated, ATP-Mn CNP showed better antitumor effects compared with TPP-Mn CNP (Fig. 6c). This trend was further validated by individual tumor growth trajectories and tumor images (Figs. S25–26). To interrogate the immunomodulatory effects of these treatments, mice were sacrificed on day 18 and tumor-infiltrating immune cells were analyzed by flow cytometry (Fig. S27). As indicated, compared with TPP-Mn CNP treatment, ATP-Mn CNP treatment significantly increased the percentage of CD80^+^ macrophages and markedly decreased the percentage of CD206^+^ macrophages within tumor tissues (Fig. 6d and e). While TPP-Mn CNP treatment did not substantially alter the M1/M2 macrophage ratio in tumors, ATP-Mn CNP treatment led to a significant elevation of the M1/M2 ratio (Fig. 6f). In addition, ATP-Mn CNP treatment also significantly increased the percentage of CD80^+^ DCs within tumor tissues compared with TPP-Mn CNP (Fig. 6g). Due to the pivotal role of T cells in antitumor immunity, we further investigated the tumor infiltration of CD3^+^, CD4^+^, and CD8^+^ T cells. The results showed that TPP-Mn CNP modestly increased the percentages of CD3^+^ and CD8^+^ T cells, whereas ATP-Mn CNP more markedly enhanced the proportions of CD3^+^, CD4^+^, and CD8^+^ T cells (Fig. 6h–j). In addition, we further evaluated the activation status of both CD8^+^ and CD4^+^ T cells by analyzing the expression of IFN-γ and CD69 on these cells (Fig. 6k–l and S28). As shown, ATP-Mn CNP treatment significantly enhanced IFN-γ and CD69 expression in both CD8^+^ and CD4^+^ T cells, indicating robust T cell activation. In addition, ATP-Mn CNP treatment also promoted infiltration of natural killer (NK) cells and B cells, indicating broad activation of the antitumor immune responses in the tumor microenvironment (Fig. S29). Examination of TDLNs revealed that ATP-Mn CNP treatment increased CD80 expression while reducing CD206 levels on macrophages, resulting in a significantly higher M1/M2 ratio compared to TPP-Mn CNP, consistent with systemic immune activation (Fig. 6m–o). In addition, H&E staining of tumor tissues from various treatment groups in the MC38 model was conducted to evaluate tumor morphology (Fig. S30). Compared with the control and TPP-Mn CNP groups, ATP-Mn CNP-treated tumors showed marked tumor cell death, showing the pathological evidence for its antitumor efficacy. These findings suggest that ATP-Mn CNP could elicit robust antitumor immune responses by driving macrophage polarization toward a proinflammatory phenotype, facilitating DC maturation, and increasing infiltration of T cells, B cells, and NK cells.

### 2.6 The combination of ATP-Mn CNP and anti-PD-1 antibody elicits strong and durable antitumor immunity

**Fig. 7:**
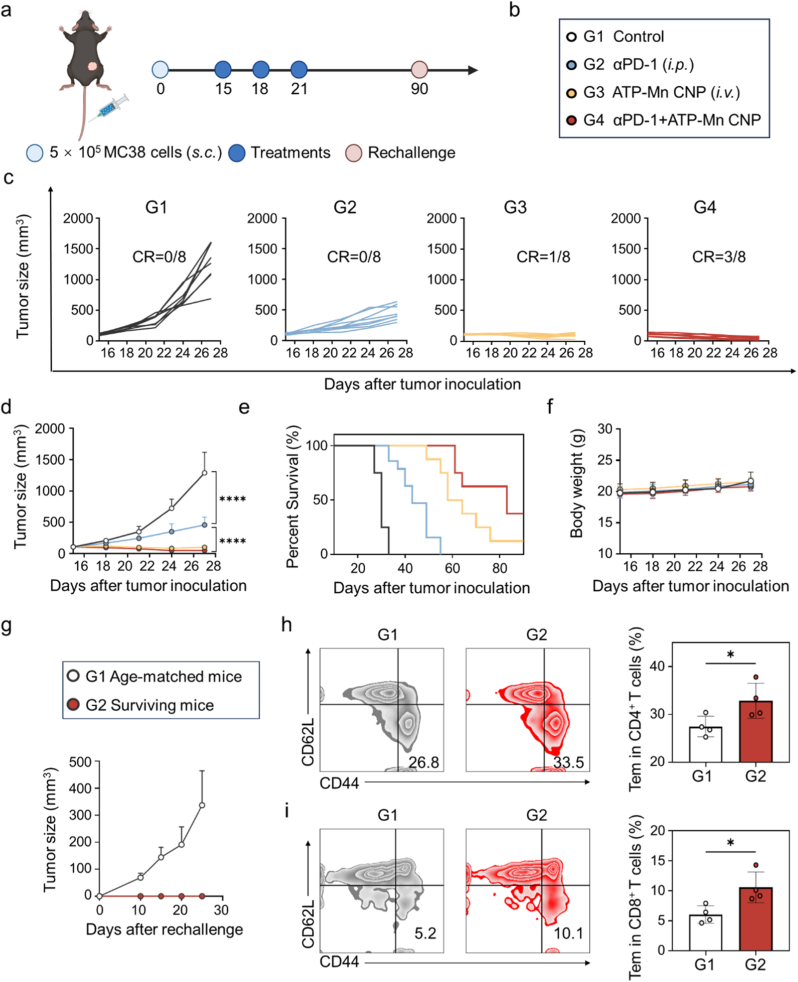
**Combination of ATP-Mn CNP and anti-PD-1 antibody inhibits tumor growth in the MC38 model.** (a-b) Schematic illustration of the treatment process. (c) Survival rate of mice in the indicated groups (n = 8). (d) Tumor size curves of MC38 tumor-bearing mice in various treatment groups. (e) Spider diagram of tumor growth curves of individual mouse from various groups. (f) Body weight change of MC38-bearing mice (n = 8). (g) Tumor size curves of MC38 tumor-rechallenge mice in various treatment groups. (h) Percentages of CD44^+^CD62L^−^CD4^+^T cells in the spleens of mice from different groups (n = 4). (i) Percentages of CD44^+^CD62L^−^CD8^+^T cells in the spleens of mice from different groups (n = 4). Data are presented as the mean ± SD. Statistical significance is indicated as ∗P < 0.05, ∗∗P < 0.01, ∗∗∗P < 0.001, and ∗∗∗∗P < 0.0001.

Building upon our previous findings that ATP-Mn CNP elicited potent immune activation in the MC38 tumor model, we next investigated whether their antitumor efficacy could be further augmented through combination with immune checkpoint blockade (ICB). Among ICB strategies, antibodies targeting programmed cell death protein 1 (PD-1) are widely employed in clinical oncology to relieve T-cell suppression [54,55]. MC38 tumor-bearing C57BL/6 mice were randomly assigned to four treatment groups: PBS control (G1), αPD-1 monotherapy (G2, *i.p.*), ATP-Mn CNP monotherapy (G3, *i.v.*), and the αPD-1 plus ATP-Mn CNP combination (G4) (Fig. 7a and b). The dosage and administration frequency of αPD-1 were set as previously reported [23,43,56]. Treatments were administered according to optimized schedules, and tumor progression was monitored over time. As indicated, compared with αPD-1 monotherapy which achieved a 64.5% tumor growth inhibition rate, ATP-Mn CNP monotherapy and the combination therapy showed 92.1% and 96.3% tumor growth inhibition rates, respectively (Fig. 7c and d). More significantly, compared with 1/8 tumor eradication achieved by ATP-Mn CNP monotherapy, the combination of αPD-1 and ATP-Mn CNP eradicated 3/8 of the tumors. Mice survival analysis demonstrated that ATP-Mn CNP treatment extended lifespan compared with PBS treatment, and combination therapy further enhanced survival benefits. The median survival time of mice treated with PBS, αPD-1, ATP-Mn CNP, and combination therapy were 30, 42, 61, and 83 days, respectively, indicating a synergistic antitumor efficacy (Fig. 7e). All treatments did not cause notable body weight loss, indicative of a favorable safety profile (Fig. 7f).

To assess whether these antitumor responses conferred durable immunity, all surviving mice, including those in the ATP-Mn CNP monotherapy group and the ATP-Mn CNP/αPD-1 combination therapy group, were rechallenged with fresh MC38 cells. Remarkably, none of the previously cured mice developed secondary tumors (Fig. 7g), confirming the establishment of long-term immunological protection. We performed the analysis of memory T cell subsets in the spleens of mice following secondary tumor rechallenge, including central memory T cells (Tcm, CD44^+^CD62L^+^) and effector memory T cells (Tem, CD44^+^CD62L^−^). Flow cytometry analysis showed that the proportion of Tcm did not show a significant difference among the treatment groups, whereas a pronounced increase in Tem was observed in the surviving mice (Fig. 7h–i and S31). Given the critical role of Tem in mediating rapid immune responses upon tumor rechallenge [57,58], these results demonstrate that ATP-Mn CNP not only potentiates immediate antitumor responses but also induces a durable systemic antitumor immunity primarily by enhancing Tem responses.

### 2.7 *In Vivo* Biosafety of ATP-Mn CNP

**Fig. 8:**
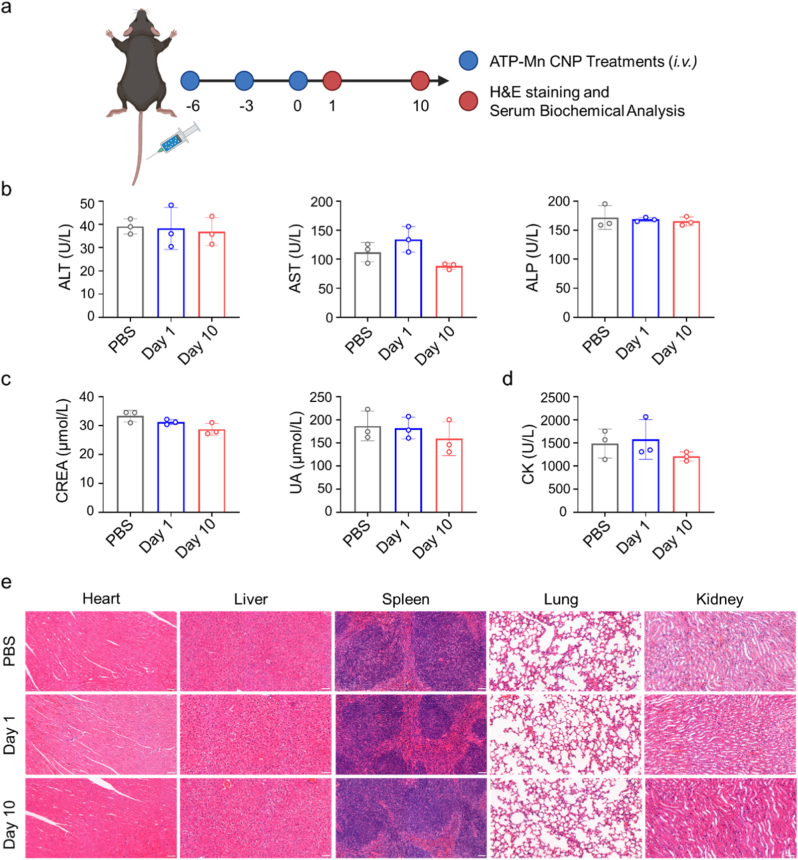
**Preliminary examination of the *in vivo* biosafety of ATP-Mn CNP.** (a) Schematic illustration of the treatment process. (b-d) Serum biochemical analysis of mice receiving different treatments on day 1 and day 10 after the last injection (n = 3). (e) Representative H&E staining images of heart, liver, spleen, lung, and kidney from the indicated groups. Scale bar = 50 μm.

The biocompatibility of nanoformulation is important for its biomedical application. We first investigated the *in vivo* organ distribution profile of ATP-Mn CNP (Fig. S32). Following intravenous administration of DiD-labeled ATP-Mn CNP, *ex vivo* organ imaging revealed that the particles were primarily localized in the liver, with modest accumulation in the lungs and tumor tissues, while accumulation in the brain remained minimal. By day 10 after consecutive dosing, most of the nanoparticles had been cleared, with only residual signals detectable in the liver. We tested the biocompatibility of ATP-Mn CNP in healthy C57BL/6 mice (Fig. 8a). We evaluated the systemic safety of ATP-Mn CNP through serum biochemical analyses at day 1 and day 10 post-treatment. Key indicators including alkaline phosphatase (ALP), aspartate aminotransferase (AST), alanine aminotransferase (ALT), creatinine (CREA), uric acid (UA), and creatine kinase (CK) were measured to evaluate liver, kidney, and cardiac function (Fig. 8b–d). The results showed that all measured parameters were within normal reference ranges, indicating that ATP-Mn CNP did not induce organ toxicity. In addition, H&E staining of major organs (heart, liver, spleen, lung, and kidney) showed no obvious tissue damage or inflammatory response (Fig. 8e). These results demonstrate that ATP-Mn CNP exhibits good biocompatibility under the dosing regimen used in this study.

## 3 Conclusions

We report the design of ATP-Mn coordination nanoparticles (ATP-Mn CNP) that exploits the dual role of ATP as both a structural ligand and an immunomodulatory signal to activate the cGAS-STING pathway. Unlike conventional metal-ligand systems, ATP confers unique bioactivity by synergizing with Mn^2+^ to amplify cGAS-STING signaling while simultaneously driving coordination-driven nanoparticle assembly. ATP-Mn CNP promotes dendritic cell maturation, reprograms tumor-associated macrophages toward a pro-inflammatory phenotype, and elicits robust type I IFN and cytokine responses, thereby enhancing antitumor immunity *in vitro* and *in vivo*. More impressively, the combination of ATP-Mn CNP and anti-PD-1 antibody achieved complete tumor eradication in almost 40% of mice, further consolidating the effectiveness of the simple nanoagonist-based design. To advance the platform, future studies can focus on elucidating the impact of ATP on immunometabolic networks. In summary, capitalizing on the abundance and cost-effectiveness of ATP, the ATP-amplified STING activation strategy circumvents the drawbacks of free Mn^2+^, thereby offering a robust and effective platform for cancer immunotherapy.

## CRediT authorship contribution statement

**Si-Yao Han:** Writing – original draft, Visualization, Methodology, Investigation, Formal analysis, Data curation. **Sui-Juan Zheng:** Validation, Methodology, Data curation. **Jia-Qi Luo:** Validation, Supervision. **Hui-Han Yu:** Methodology, Data curation. **Xiao-Yue Liu:** Methodology, Data curation. **Jin-Zhi Du:** Writing – review & editing, Supervision, Resources, Project administration, Funding acquisition.

## Ethics approval and consent to participate

All animal experiments were carried out following the Guidelines of Institutional Animals at the South China University of Technology and complied with all relevant ethical regulations (2022041).

## Declaration of competing interest

The authors declare no competing financial interests or personal relationships that could have appeared to influence the work reported in this paper.

## Data Availability

All relevant data are available with the article and its Supplementary Information files, or available from the corresponding authors upon reasonable request.
