# Supplementary material for: ATP-fueled STING activation of manganese coordinated nanoagonist to boost antitumor immunity
Source: Bioact Mater. 2026 Feb 10;61:107–20. doi: 10.1016/j.bioactmat.2026.02.012 (PMC12914834; doi:10.1016/j.bioactmat.2026.02.012)
Supplement: Multimedia component 1 [file mmc1.docx]

**Supporting Information for**

**ATP-Fueled STING Activation of Manganese Coordinated Nanoagonist to Boost Antitumor Immunity**

**Materials and Methods**

**Materials**

Manganese chloride tetrahydrate were purchased from Heowns Biochem Technologies, LLC (Tianjin, China). Adenosine 5'-triphosphate disodium salt (ATP), sodium tripolyphosphate (TPP), 1,2-Dioleoyl-sn-Glycero-3-Phosphate (DOPA), and 1,2-Dioleoyl-sn-glycero-3-phosphocholine (DOPC) were purchased from Macklin. Cholesterol and 1,2-diastearoyl-sn-glycero-3-phospho-ethanolamine-N- [amino (polyethylene glycol)2000] were purchased from Aladdin. Granulocyte-macrophage colony-stimulating factor (GM-CSF), inter-leukin-4 (IL-4), and M-CSF were obtained from PeproTech. Roswell Park Memorial Institute (RPMI)-1640, Dulbecco’s modified Eagle’s medium (DMEM), and fetal bovine serum (FBS) were purchased from Gibco. 1,1'-dioctadecyl-3,3,3',3'-tetramethylindodicarbocyanine,4-chlorobenzenesulfonate salt (DiD), Lyso-Tracker Red, and 4,6-diamino-2-phenyl indole (DAPI) were purchased from Beyotime. Primers were purchased from IGE Biotech Co., Ltd. (Guangzhou, China). Red blood cell (RBC) lysis was obtained from Beyotime Biological Co. The collagenase type IV was obtained from Sigma, and DNase I was obtained from Roche. ELISA kits were purchased from Dakewei and Multi sciences.

**Cell lines and animals**

B16F10 melanoma cell lines and MC38 cancer cell lines were originally obtained from the American Type Culture Collection (ATCC), and cell lines were cultured in RMPI 1640 medium or DMEM (Gibco) containing 10% (v/v) FBS (Gibco) and 1% (v/v) penicillin/streptomycin (Invitrogen) at 37 °C in 5% CO_2_. L929-ISRE cell lines were cultured in 10% (v/v) FBS and 1% (v/v) penicillin/streptomycin at 37 ℃ in 5% CO_2_. Male C57BL/6 mice were purchased from GemPharmatech Co., Ltd. Tumors were implanted when the mice were 6-8 weeks old. The mice were maintained under specific pathogen-free (SPF) conditions in the Laboratory Animal Center of the South China University of Technology. All animal experiments were carried out following the Guidelines of Institutional Animals at the South China University of Technology and complied with all relevant ethical regulations (2022041).

**Synthesis of ATP-Mn CNP and TPP-Mn CNP**

ATP-Mn CNP was prepared in two steps. Briefly, an aqueous solution of ATP was added to 10 mL of a mixture of Triton X-100 and 1-hexanol in cyclohexane and stirred vigorously for 30 min in the presence of DOPA (CHCl_3_). An aqueous solution of MnCl_2_ was added to 10 mL of a mixture of Triton X-100 and 1-hexanol in cyclohexane and stirred vigorously for 15 min. The MnCl_2_-containing microemulsion was added dropwise to the ATP-containing microemulsion and stirred vigorously for 30 min at room temperature. After the addition of 20 mL ethanol, the ATP-Mn core was obtained by centrifugation at 14000 rpm. The ATP-Mn core was washed twice with 50% (v/v) tetrahydrofuran (THF)-ethanol and finally redispersed in THF. The ATP-Mn core was prepared by adding a THF solution (0.2 mL) of DOPC, cholesterol and DSPE-PEG2000 (2:2:1) and the ATP-Mn core to 1 mL of 30% (v/v) ethanol-water at room temperature. Organic reagents are evacuated under a nitrogen stream. The preparation of TPP-Mn CNP was consistent with the above. The particle size and zeta potential of ATP-Mn CNP and TPP-Mn CNP were measured by dynamic light scattering (DLS) on Anton Paar Litesizer 500 Particle Analyzer (Anton Paar Corporation, Austria). The composition of ATP-Mn CNP was determined by inductively coupled plasma mass spectrometry (ICP-MS) for Mn content and by a commercial ATP assay kit (Beyotime) for ATP content. The morphology of ATP-Mn CNP and TPP-Mn CNP was observed by a transmission electron microscope (TEM Talos L120c, Talos F200x).

**Bone-marrow-derived cell (BMDC or BMDM) extraction and induction**

Tibias and femurs were removed from C57BL/6 mice (male, 6-8 weeks old), and both ends of the bones were cut with scissors. After washing with PBS, bone marrow cells were flushed out of the tibias and femurs with a syringe needle. After centrifugation, red blood cells (RBCs) were lysed with a diluted RBC lysis buffer. For BMDC induction, bone marrow cells were dispersed in complete RPMI-1640 containing inactivated FBS (56 °C, 30 min), 20 ng/mL GM-CSF, and 5 ng/mL IL-4. Then, the cells were seeded with a density of 5 × 10^5^ per well in 24-well plates and incubated at 37 °C in 5% CO_2_. For BMDM induction, bone marrow cells were dispersed in complete DMEM containing 10% FBS and 10 ng/mL M-CSF. The culture medium was replaced with fresh media containing cytokines every 2-3 days, and immature BMDCs or BMDMs can be obtained on day 5 or 6.

**Determination of Mn^2+^ release from ATP-Mn CNP**

For the measurement of Mn^2+^ release, 2 mL of ATP-Mn CNP solution was added to the dialysis bag (MWCO = 2000 Da) and then placed into centrifuge tubes with 20 mL of buffer solution (pH 7.4 or 5.7) as the external solution. The tube was shaken at 37 °C in a water bath. At determined time points, 5 mL of the release solution was collected each time, and 5 mL of fresh buffer solution was added. The content of Mn^2+^ in the solution was detected by inductively coupled plasma mass spectrometry (ICP-MS, iCAP 7200 Duo, Thermofisher Scientific).

**Cellular uptake and intracellular localization assay**

BMDMs or BMDCs were plated with a density of 2 × 10^5^/well on a cell climbing sheet in 24-well plates and incubated at 37 °C in 5% CO_2_ overnight. To investigate the mechanism of endocytosis, cells were pretreated with different inhibitors of endocytosis, amiloride (400 μM), Chlorpromazine (20 μM), Dynasore (80 μM) and Genistein (1 mM) for 1 h. Then, DiD-labeled ATP-Mn CNP was coincubated with the cells for 0, 3, 6, 12, 24 h. After being washed with PBS, the cells were collected for flow analysis. For the confocal experiment, after treating with DiD-labeled ATP-Mn CNP, the cell climbing sheets adhering to BMDMs were stained with Lysotracker Red. After washing with PBS, the cells were stained with DAPI and washed with PBS three times. Finally, the cell climbing sheets adhering to BMDMs were flipped upside down on the glass slides. Lysosome localization was detected by Lysotracker Red (Beyotime Biological Co.) according to the manufacturer’s guidelines. The fluorescence imaging was performed by laser confocal microscopy (Nikon, A1+, Japan).

***In vitro* cell viability and hemolysis assay**

To estimate cell viability treated with ATP-Mn CNP, BMDCs or BMDMs were plated with a density of 1 × 10^4^/well in 96-well plates and cultured overnight. After coincubating with different concentrations of ATP-Mn CNP for 24 h, 3-[4,5-dimethylthiazol-2-yl]-2,5-diphenyl tetrazolium bromide (MTT, 5%, 10 μL) was added in 96-well plates at 37 °C in 5% CO_2_ for 4 h. After discarding the supernatant, 100 μL of DMSO was added into each well. The plate was shaken for 5 min at room temperature, and then, the absorbance at 490 nm was measured by a microplate reader (Biotek). For hemolysis assay, 1 mL of mouse blood containing EDTA was added in 9 mL of PBS (pH = 7.4) and mixed gently. The blood was washed four times and dispersed in PBS containing different concentrations of ATP-Mn CNP or H_2_O. After incubating at 37 °C for 1 h, the samples were centrifuged and photographed, and the supernatant was collected in 96-well plates for the measurement at OD540 nm by microplate reader (Biotek).

**mRNA transcriptome sequencing and RT-qPCR Analysis**

After treatment with ATP + MnCl_2_ (50 μM ATP + 150 μM MnCl_2_, physical mixture) or ATP-Mn CNP (150 μM Mn/50 μM ATP), mRNA from BMDMs was collected for gene expression analysis by Novagene Company. The fold change was calculated by comparing it with the average normalized gene in the PBS group. For the RT-qPCR analysis, BMDMs were incubated with PBS, TPP-Mn CNP, and ATP-Mn CNP for 18 h. The total RNA was isolated by Trizol, and the mRNA expression level of *Ifnb1*, *Isg20*, *Isg15*, *Cd206*, *Arg1*, *Nos2*, *Cxcl10* and *Il1b* was analyzed by quantitative real-time PCR (RT-qPCR). Quantitative real-time PCR was performed with SYBR Green incorporation on the Light Cycler 96 System (Roche), and the data were presented as an accumulation index. Primer sequence information in the RT-qPCR assay is displayed in **Table S1**.

***In vitro* activation assay of BMDCs and BMDMs**

BMDCs or BMDMs were incubated with PBS, ATP + MnCl_2_ (50 μM ATP + 150 μM MnCl_2_, physical mixture) or ATP-Mn CNP (150 μM Mn/50 μM ATP) for 24 h. Cells were washed once and dispersed in PBS containing 0.2% bovine serum albumin (BSA). Cells were treated with blocking solution (α-CD16/32, 4 °C, 20 min) and were labeled by fluorescently conjugated antibodies including PerCP-Cy5.5-anti-CD11c (Biolegend), PE-CF594-anti-CD80 (Biolegend) or FITC-CD80 (Biolegend), APC-anti-CD86 (Biolegend), PE-anti-CD (Biolegend) and PE-Cy7-F4/80 (Biolegend). After being washed in PBS containing 0.2% BSA, the mean fluorescence intensity of the costimulatory molecules (CD80, CD86) was assessed by flow cytometry.

**Determination of cytokines by ELISA assay**

Samples from the culture supernatant of BMDMs, BMDCs and tumor were collected and first subjected to concentration determination by the BCA protein concentration assay kit (Beyotime, P0012). Cytokines such as TNF-α and IFN-β were tested by ELISA kits according to the manufacturer’s instructions. The readouts were obtained by using a microplate reader (Biotek) at 450 nm.

**Type I IFN bioassay**

BMDMs or BMDCs were treated with ATP + MnCl_2_ (50 μM ATP + 150 μM MnCl_2_, physical mixture) or ATP-Mn CNP (150 μM Mn and 50 μM ATP) for different points. Then, the culture supernatant of BMDCs or BMDMs was added to L929-ISRE cell lines in 96-well plates (2 × 10^4^ per well). After 4 h incubation, the cells were lysed, and the luciferases were lysed and detected with firefly luciferase reporter gene cell lysate (Beyotime Biological Co, RG126S) and detection kit (Beyotime Biological Co, RG058S) by a microplate reader (Biotek). This functional assay reflects the overall activity of type I interferons (including IFN-α and IFN-β) through ISRE-driven luciferase expression.

***In vitro* killing assay of B16-Luc and MC38-Luc cells by BMDMs**

BMDMs were incubated with PBS, ATP + MnCl_2_ (50 μM ATP + 150 μM MnCl_2_, physical mixture) or ATP-Mn CNP (150 μM Mn/50 μM ATP) for 24 h. Cells were collected and coincubated with B16-Luc/MC38-Luc cells (4:1) in 24-well plates for 24 h. Then, the cells were lysed, and the luciferases were lysed and detected with firefly luciferase reporter gene cell lysate (Beyotime Biological Co, RG126S) and detection kit (Beyotime Biological Co, RG058S) by a microplate reader (Biotek).

***In vitro* phagocytosis assay**

For flow cytometric analysis, BMDMs separated from C57BL/6 mice were seeded in 24-well plates at 5×10^4^ cells per well for attachment. B16F10 cells were labeled with live cell proliferation dye CFSE (Beyotime, C1031) then co-culture with BMDMs in a ratio of 4:1, with adding of ATP + MnCl_2_ or ATP-Mn CNP. After 24 hours, all cells were collected and incubated with anti-CD16/32 antibody for 20 min at 4 °C, followed staining with APC anti-F4/80 antibody for 30 min. After that, cells were washed 3 times with PBS and re-suspended in PBS containing 0.2% BSA for flow cytometric analysis. Dead cells were excluded by DAPI. The percentage of phagocytosis was determined by the percentage of dual positive BMDMs (CFSE^+^ APC^+^) within total BMDMs population (APC^+^).

***In vitro* intracellular Mn content detection**

BMDMs were incubated with PBS, ATP-Mn CNP (150 μM Mn) and TPP-Mn CNP (150 μM Mn) for 24 h, respectively. Cells were washed twice and dispersed in PBS. The intracellular content of Mn was quantified by ICP-MS, (iCAP 7200 Duo, Thermofisher Scientific).

**Western blot analysis**

All cell lysates and Prestained Protein Ladder were separated by 10% SDS-polyacrylamide gel electrophoresis and then transferred into nitrocellulose membranes. After blocking with 5% bovine serum albumin (BSA) in Tris-buffered saline containing 0.1% Tween-20 for 2 h at room temperature, the membranes were incubated with specific anti-STING (1;2000), anti-p-STING (1;2000), anti-IRF3 (1:2000), anti-p-IRF3 (1;2000), anti-TBK1 (1:2000), anti-p-TBK1 (1;2000) and anti-β-actin (1:2000) from Cell Signaling Technology overnight at 4 °C. Then the membranes were washed and incubated with horseradish peroxidase-conjugated secondary antibodies. Developed using chemiluminescence imaging and quantified grayscale values using ImageJ software.

***In vivo* antitumor studies**

For the *in vivo* treatment immunoassay experiment, C57BL/6 mice were injected subcutaneously with 5 × 10^5^ B16F10 cells or 1 × 10^6^ MC38 cells in 50 μL of PBS into the right hind limb. On day 6 (B16F10 tumor) or 10 (MC38 tumor) after the inoculation, animals with tumors of ∼100 mm^3^ were randomly divided into three groups and treated respectively with PBS, ATP + MnCl_2_ and ATP-Mn CNP (or PBS, ATP-Mn CNP and TPP-Mn CNP) administration every 3 days, dosage for all free drugs and particles is 2 mg/kg Mn. Tumor sizes and body weights were recorded. The tumor volumes were measured with a caliper and calculated as follows: tumor volume (V) = *a* × *b*^2^/2 (a is the greatest longitudinal diameter (length), and b is the greatest transverse diameter (width)). Mice were sacrificed and defined as dead when their tumor volume reached the maximum allowed limit of 1500 mm^3^. The average inhibition rates were calculated as follows: the average inhibition rates = (1 - *c*/*d*) × 100% (c represents the tumor volume of each group, d represents the tumor volume of the PBS group on the same day). For the MC38 survival monitoring model, C57BL/6 mice were injected subcutaneously with 5 × 10^5^ MC38 cells in 50 μL of PBS into the right hind limb. On day 15 after the inoculation, animals with tumors of ~100 mm^3^ were randomly divided into four groups and treated respectively with PBS, αPD-1 (100 μg, *i.p.*), ATP-Mn CNP (2 mg/kg Mn, *i.v.*), and ATP-Mn CNP (2 mg/kg Mn, *i.v.*) + αPD-1 (100 μg, *i.p.*) administration every 3 days.

**Analysis of immune cells after the treatments**

At 48 h after the last treatment, tumor-draining lymph nodes, spleens, and tumors were harvested and cut into small pieces. Lymph nodes and spleen samples were filtered by 200-mesh nylon mesh and collected by centrifugation at 450g for 5 min at 4 °C. After lysing by RBC lysis buffer, washing, and centrifugation, cells were dispersed in PBS containing 0.2% BSA for flow cytometry antibodies staining. The tumor pieces were resuspended and digested in the DMEM medium containing 1 mg/mL collagenase type IV and 100 μg/mL DNase I. The whole solution was incubated in the shaker for 40 min at 37 °C. Digested cells were filtered by 200-mesh nylon mesh and then collected by centrifugation at 450 g for 5 min at 4 °C. Then, the lymphocytes were purified by adding 40% Percoll by centrifugation (800g) for 20 min. After lysing by RBC lysis buffer, washing, and centrifugation, cells were dispersed in PBS containing 0.2% BSA. One million cells were treated with blocking solution (α-CD16/32 4 °C, 20 min) and stained with flow cytometry antibodies (4 °C, 30 min). Samples were stained with antibodies as follows: (1) Spleen: APC-Cy7-CD45 (Biolegend), BV421-CD3 (Biolegend), BUV563 anti-CD4 (BD), and BUV737 anti-CD8 (BD). (2) Lymph nodes: APC-Cy7-CD45 (Biolegend), BV650-CD11b (Biolegend), PE/Dazzle 594-Gr-1 (Biolegend), PE-Cy7-F4/80 (Biolegend), PerCP/Cy5.5-CD11c (Biolegend), BV421-α-IA/IE (Biolegend), APC-CD86 (Biolegend), FITC-F4/80 (Biolegend), PE-CD206 (Biolegend). (3) Tumor1: BV421-CD45 (Biolegend), BV650-CD11b (Biolegend), BV711-CD19(Biolegend), PE-Cy7-NK1.1 (Biolegend), FITC-CD3 (Biolegend), PE/Dazzle 594-CD4 (Biolegend), AF700-CD8 (Biolegend).APC-IFN-γ (Biolegend), PE-CD69 (Biolegend). (4) Tumor2: APC-Cy7-CD45 (Biolegend), BV650-CD11b (Biolegend), PE/Dazzle 594-Gr-1 (Biolegend), PE-Cy7-F4/80 (Biolegend), PerCP/Cy5.5-CD11c (Biolegend), BV421-α-IA/IE (Biolegend), APC-CD86 (Biolegend), FITC-F4/80 (Biolegend), PE-CD206 (Biolegend).

**Biosafety assay of ATP-Mn CNP**

Healthy female WT C57BL/6 mice were intravenously injected with PBS and ATP-Mn CNP (2 mg/kg Mn, *i.v.*) every 3 days for 3 times, respectively. On day 1 or day 10 after treatments, the mice were sacrificed, and the whole blood was obtained for biochemical analysis. The tested biochemical items included alkaline phosphatase (ALP), aspartate aminotransferase (AST), alanine aminotransferase (ALT), creatinine (CREA), uric acid (UA), and creatine kinase (CK). The major organs of mice, such as the heart, liver, spleen, lungs, and kidney were obtained for hematoxylin-eosin (H&E) staining.

**Statistical analysis**

All results are displayed as mean ± standard deviation (S.D.). Tukey post-hoc tests and one-way ANOVA were used for multiple comparisons (when more than two groups were compared), and Student’s t test was used for two-group comparisons. All statistical analyses were performed with GraphPad Prism 10.4.0 software. A P-value less than 0.05 was considered as statistically significant (*P < 0.05, **P < 0.01, ***P < 0.001, ****P < 0.0001).

**Supporting Figures**


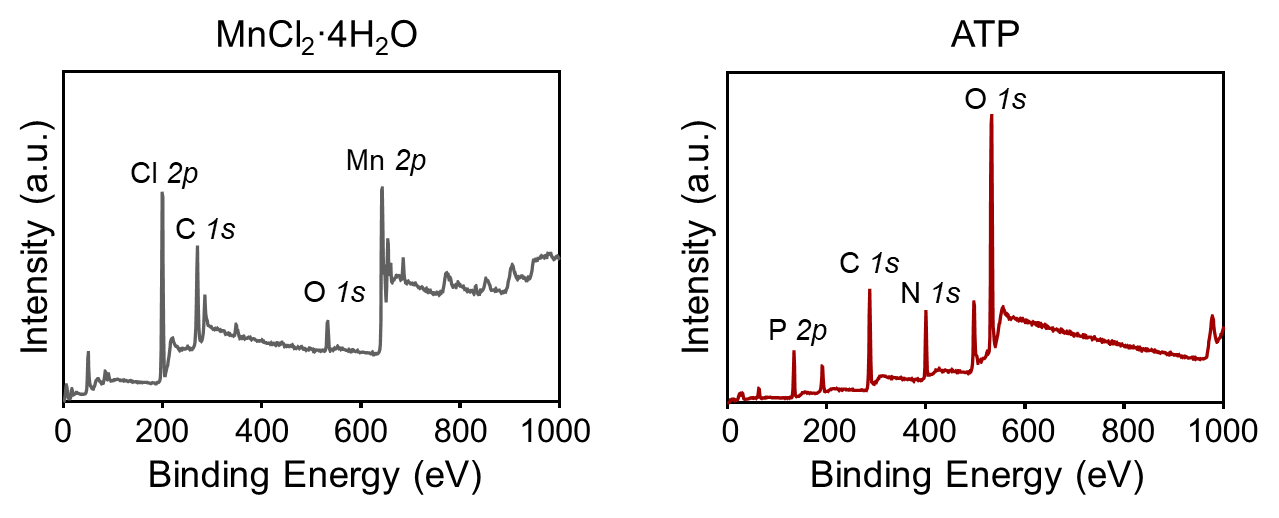


**Figure S1.** Full X-ray photoelectron spectroscopy (XPS) spectrum of MnCl_2_·4H_2_O and ATP.


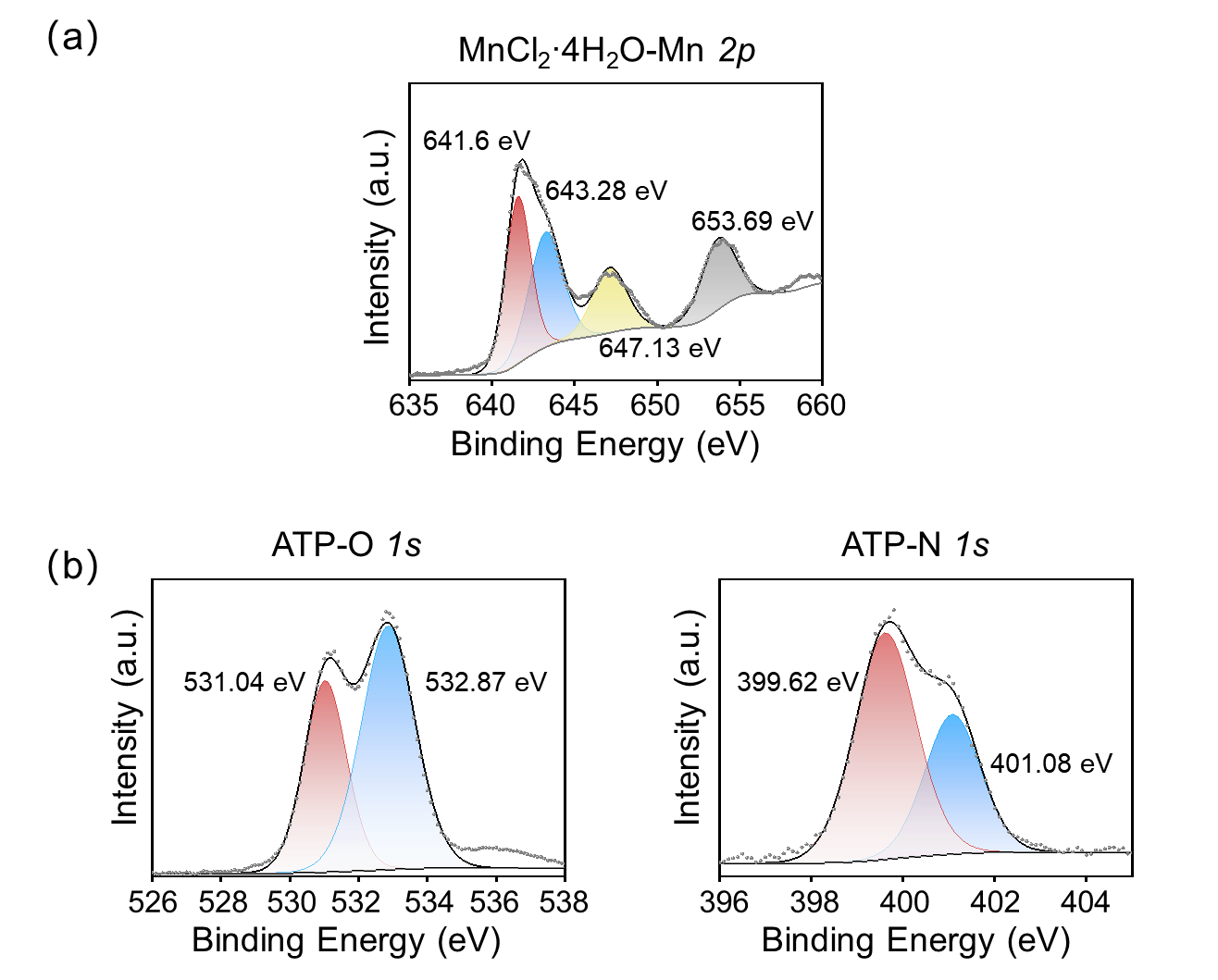


**Figure S2.** (a) High-resolution XPS spectra of Mn *2p* of MnCl_2·_H_2_O. (b) High-resolution XPS spectra of O *1s*, N *1s* of ATP. Arbitrary units (a. u.).

**
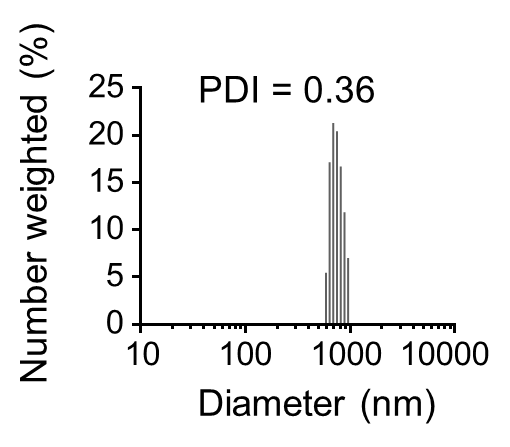
**

**Figure S3.** Size distribution of ATP-Mn complexes determined by dynamic light scattering (DLS).


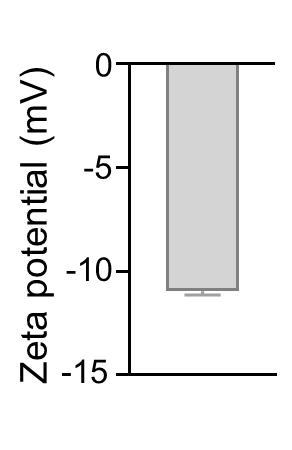


**Figure S4.** Zeta potential of ATP-Mn CNP (n = 3).


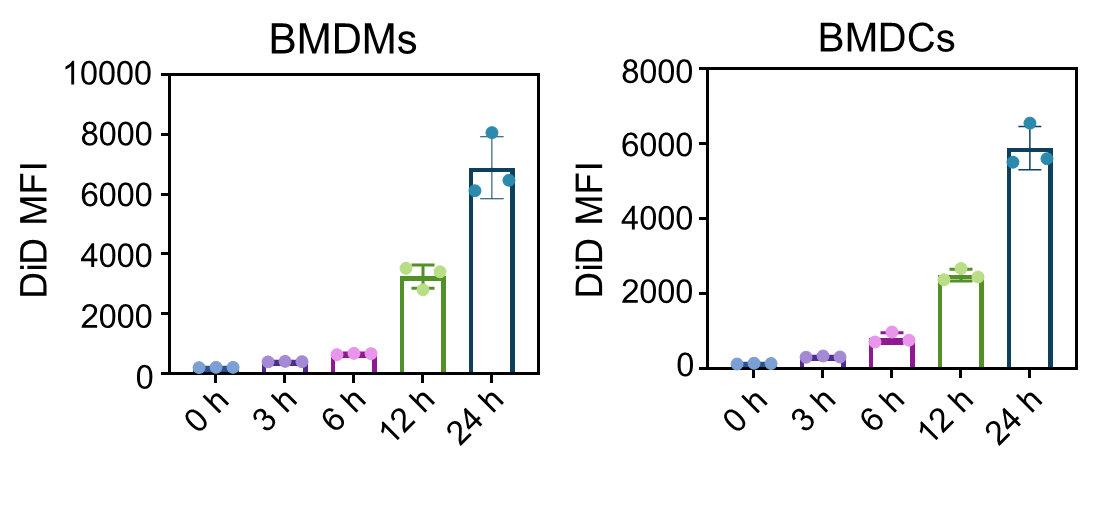


**Figure S5.** Mean fluorescent intensity (MFI) of DiD-labeled ATP-Mn CNP in BMDMs/BMDCs at different time points (n = 3).


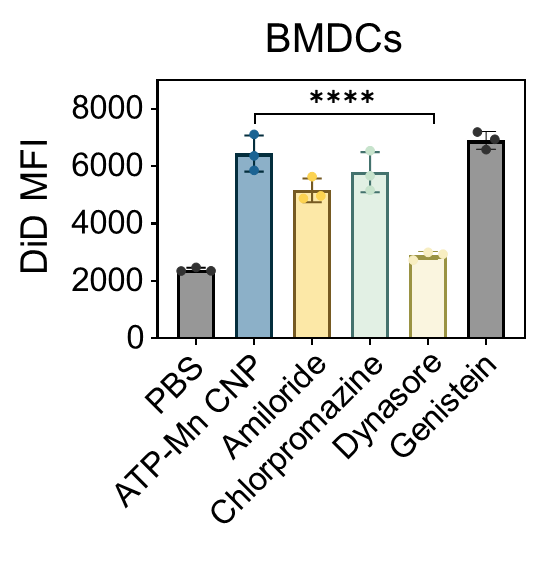


**Figure S6.** Cell uptake of ATP-Mn CNP by BMDCs after pre-treatment with different endocytosis inhibitors (n = 3).


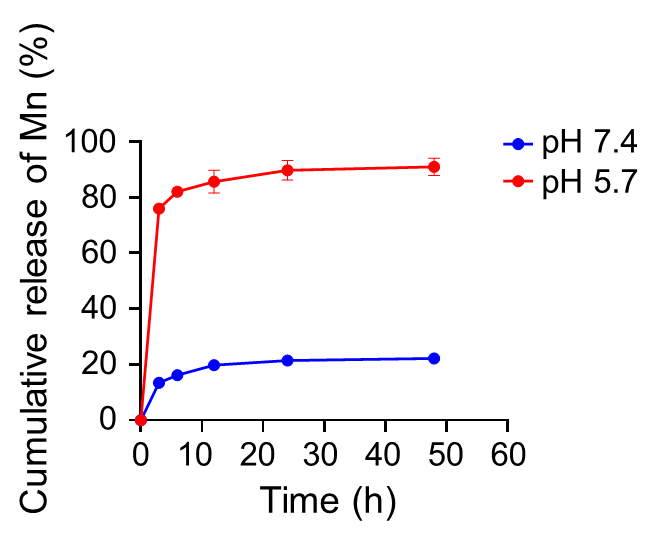


**Figure S7.** Cumulative release of Mn^2+^ from ATP-Mn CNP at pH 7.4 or 5.7 (n = 3).


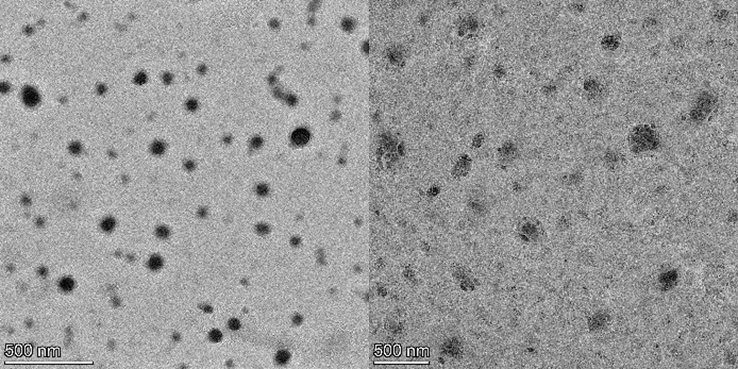


**Figure S8.** TEM observation of ATP-Mn CNP at pH 7.4 and 5.7. Scale bar = 500 nm.


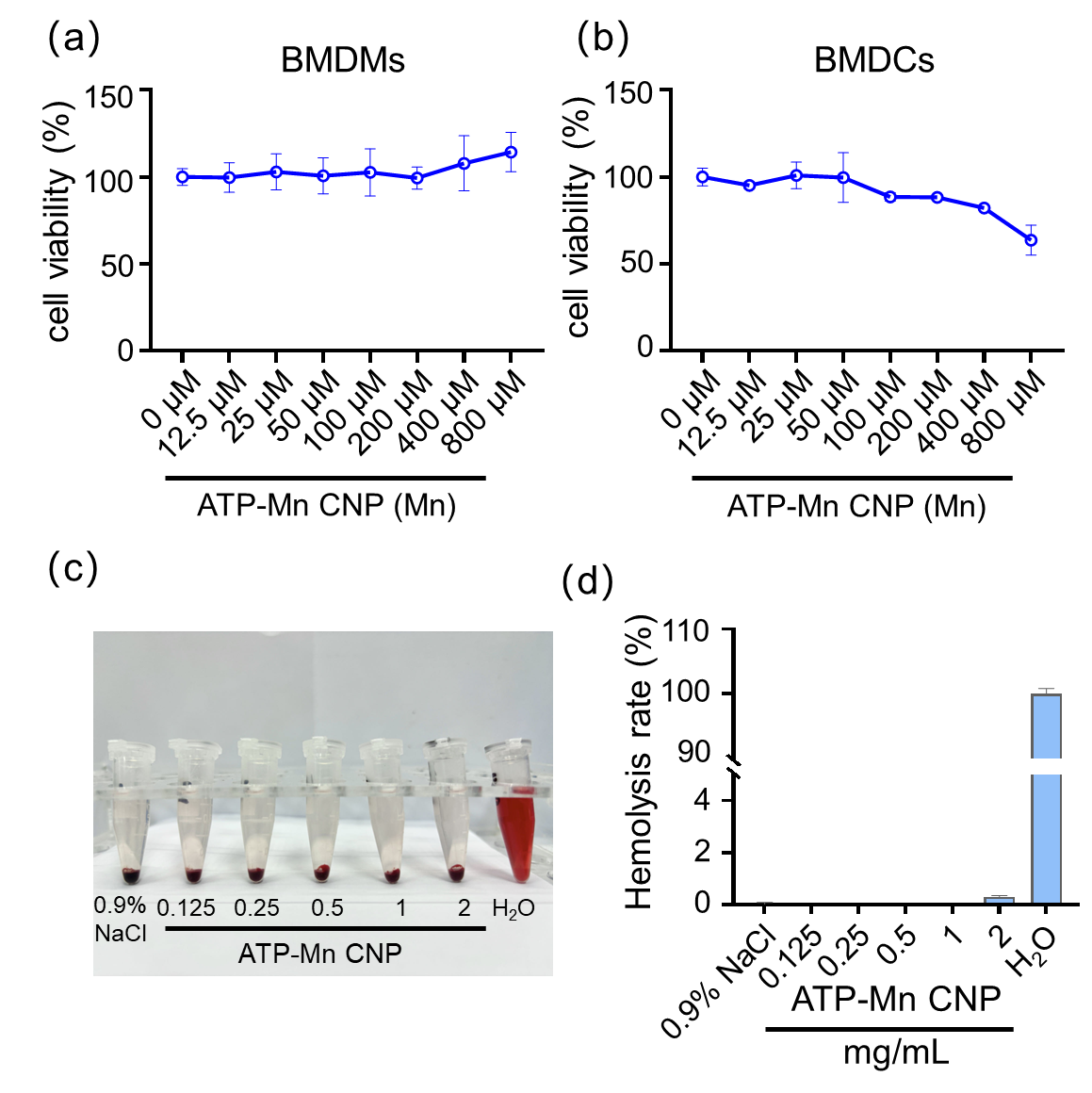


**Figure S9.** (a, b) Cell viability of BMDMs (a) and BMDCs (b) treated with different concentrations of ATP-Mn CNP (n = 3). (c, d) Hemolysis of RBCs treated with different concentrations of ATP-Mn CNP. The data are represented as means ± SD.


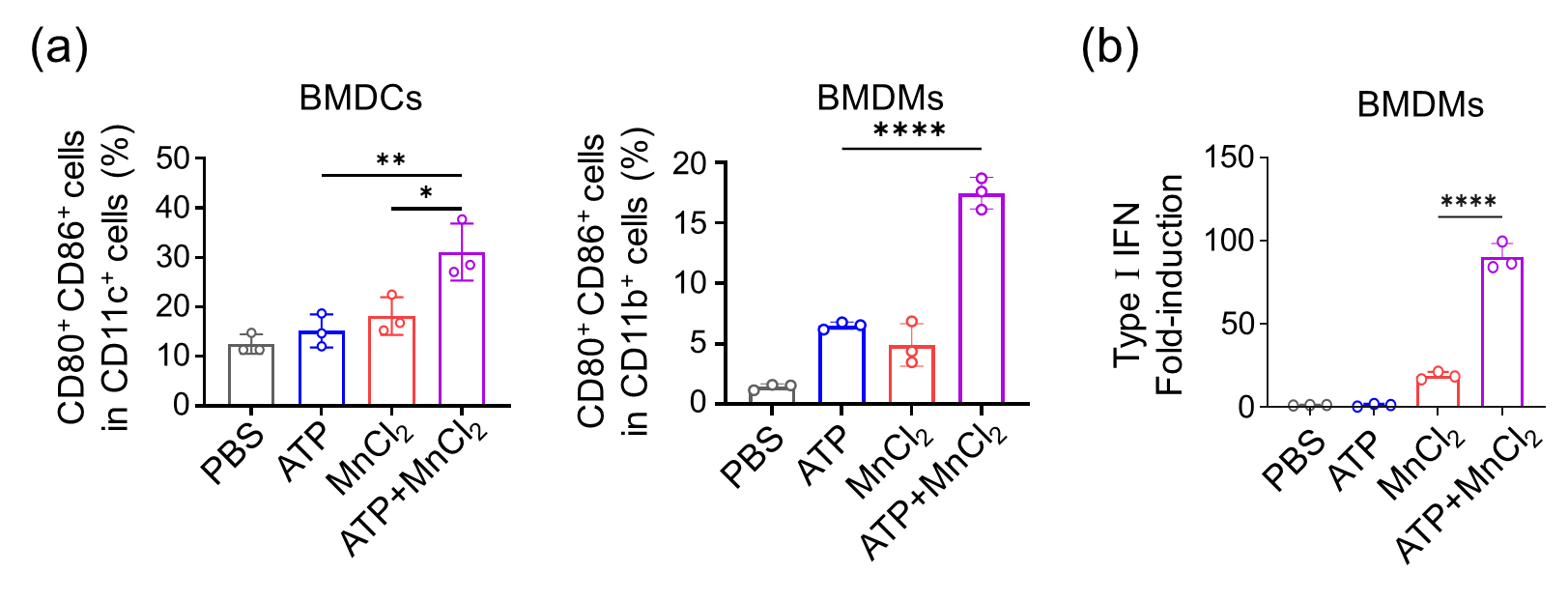


**Figure S10**. (a) Flow cytometry analysis showing the percentages of CD80⁺ CD86^+^ DCs/macrophages treated with PBS, ATP, MnCl_2_, or ATP+MnCl_2_ for 24 h (n = 3). (b) Type I IFN activity in culture supernatants from BMDMs treated with PBS, ATP, MnCl_2_, or ATP+MnCl_2_ for 24 h (n = 3).


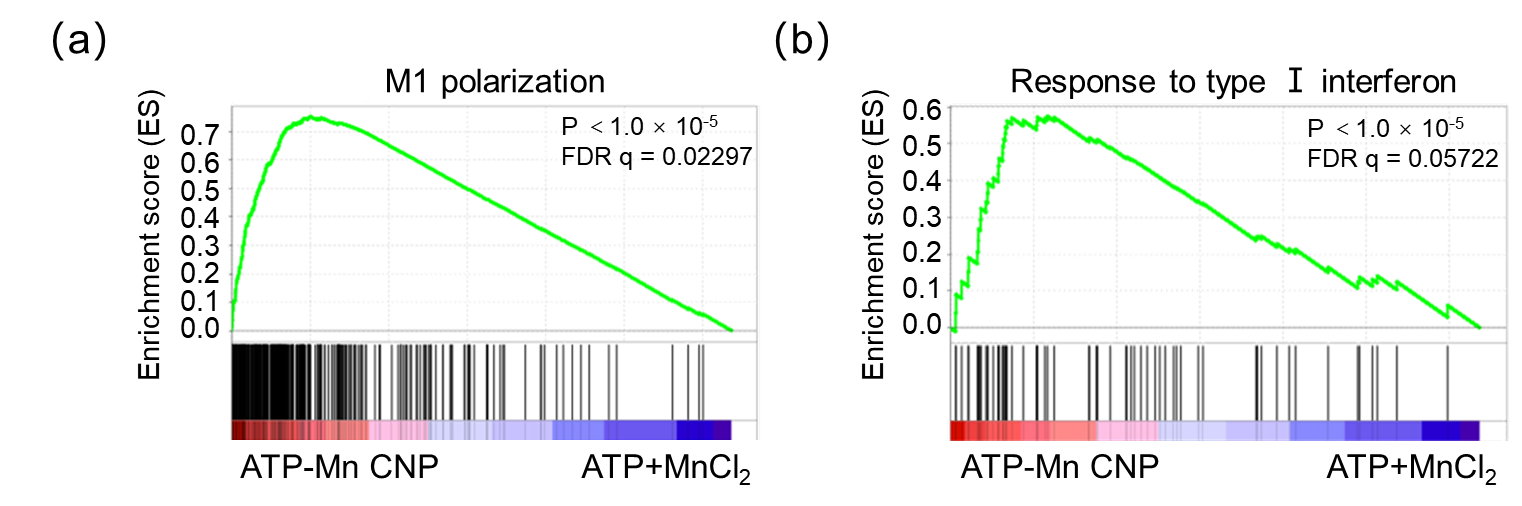


**Figure S11.** Mountain plots of the M1 polarization (a) and Response to type Ⅰ interferon (b) pathways. FDR, false discovery rate. Nominal P values are shown. Stick plots at the bottom represent the position in the ranked list of genes.


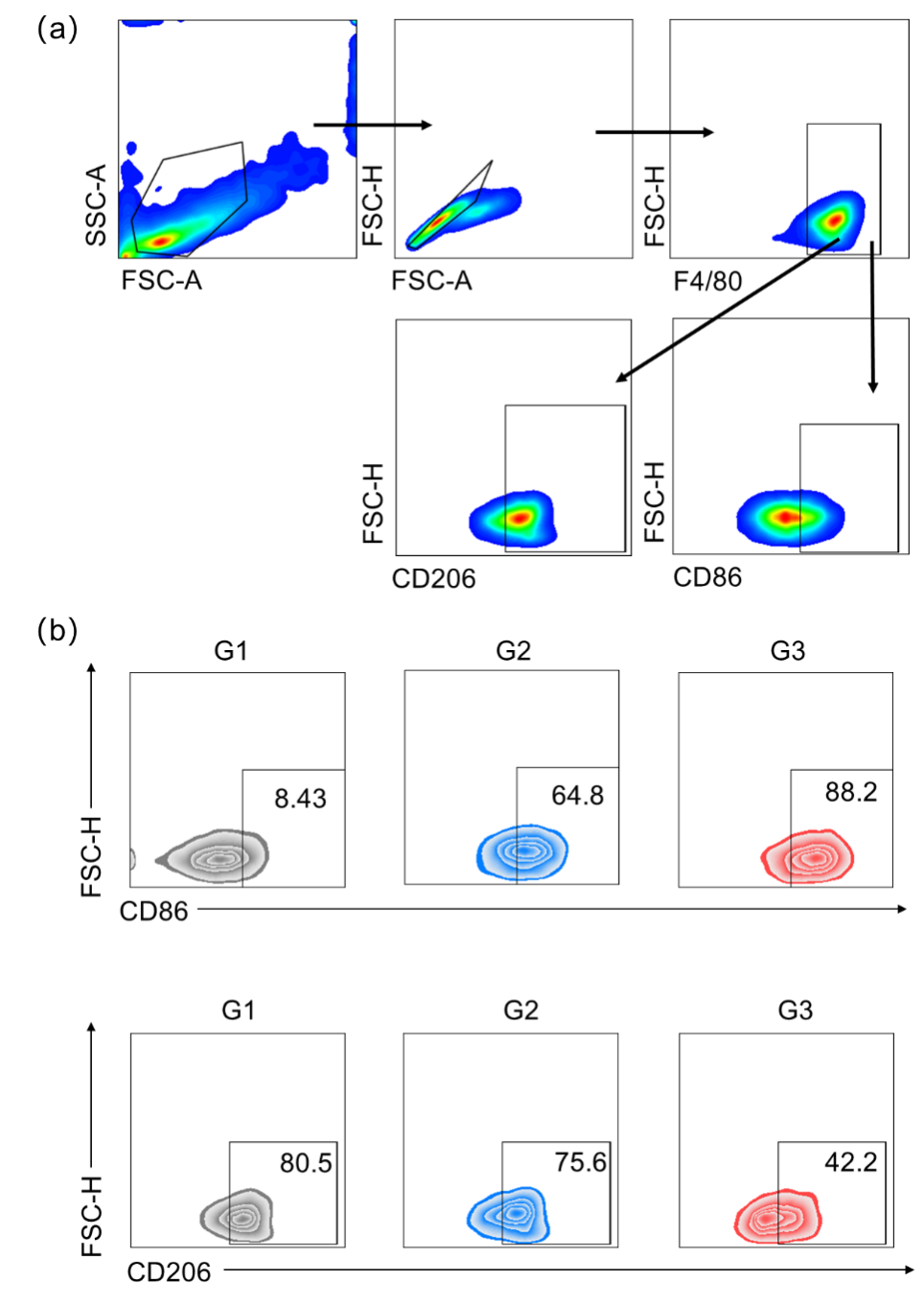


**Figure S12.** (a) Representative flow gate strategy of BMDMs repolarization. (b) Representative flow cytometry plots and percentages of CD86^+^ and CD206^+^ macrophages after different treatments (n=3).


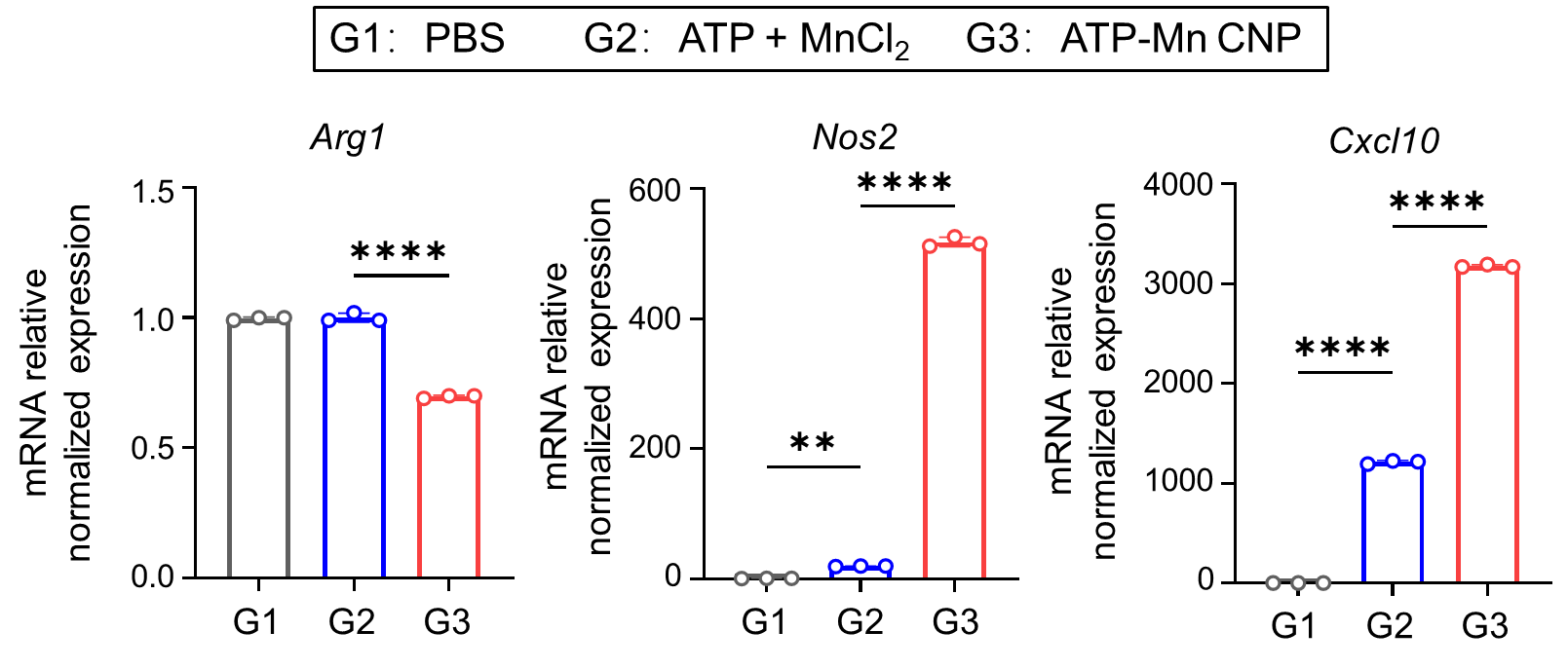


**Figure S13.** Relative mRNA expression levels of *Arg1*, *Nos2* and *Cxcl10* in BMDMs after 12 h treatment with PBS, ATP+MnCl_2_, or ATP-Mn CNP, measured by RT-qPCR (n = 3).


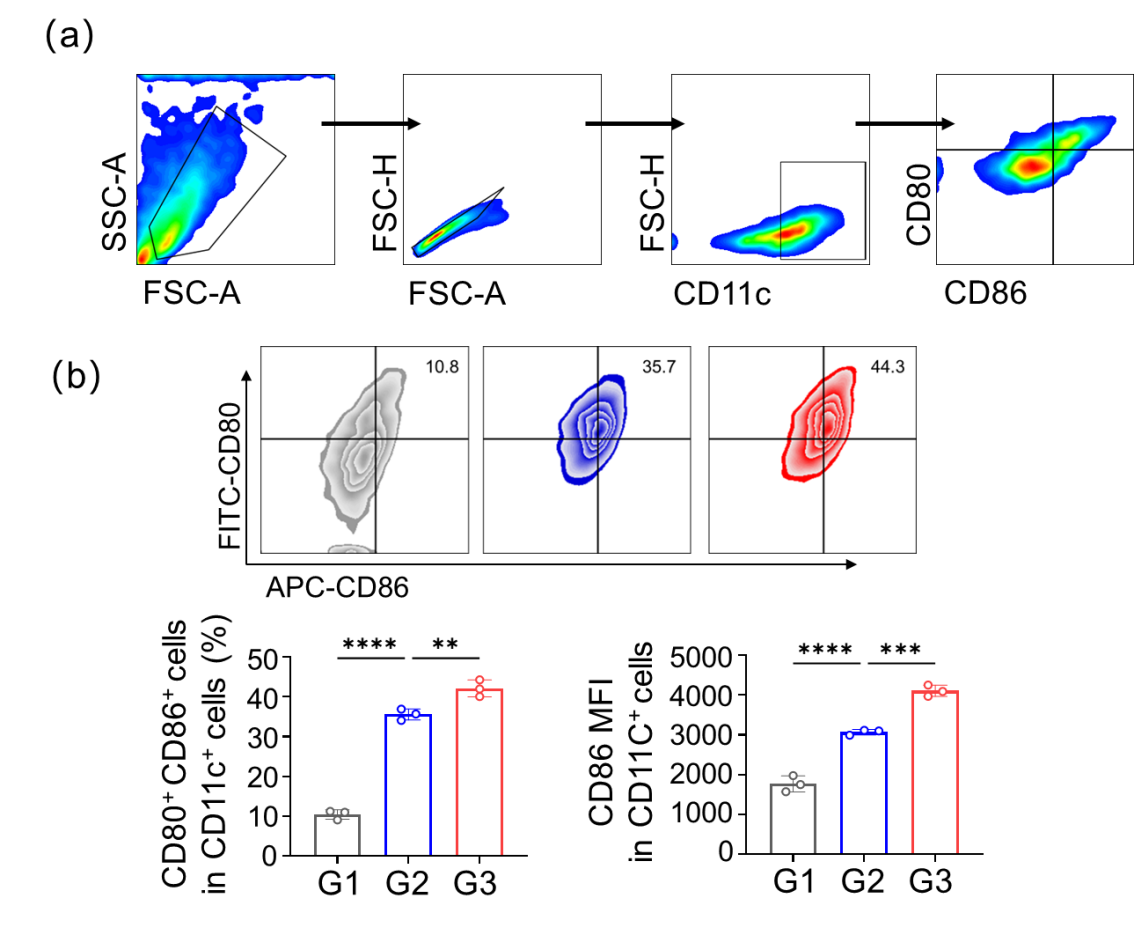


**Figure S14.** (a) Representative flow gate strategy of BMDCs activation. (b) Flow cytometry analysis and the percentage of matured DCs (CD80^+^CD86^+^) of BMDCs treated with indicated concentrations of PBS, ATP + MnCl_2_, and ATP-Mn CNP for 24 h (n = 3).


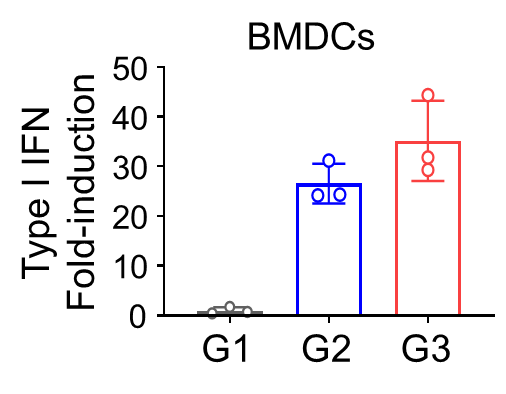


**Figure S15.** Type I IFN activity in culture supernatant from BMDCs treated with the indicated concentration of PBS, ATP + MnCl_2_ and ATP-Mn CNP for 24 h (n = 3).


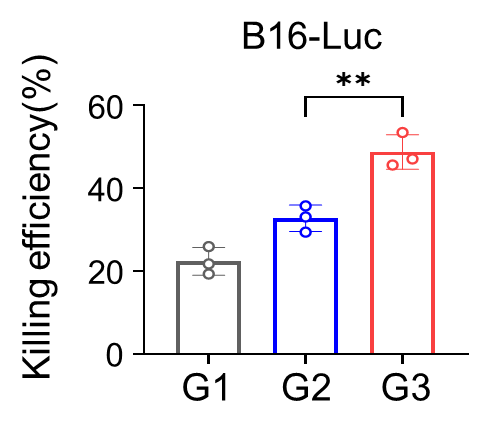


**Figure S16.** The killing efficiency of B16-luc cells by BMDMs after various treatments. BMDMs first treated with PBS, ATP + MnCl_2_ and ATP-Mn CNP for 24 h, and further co-incubated with B16-luc cells for 24 h. Then, the cells were lysed for luciferase quantification (n = 3).


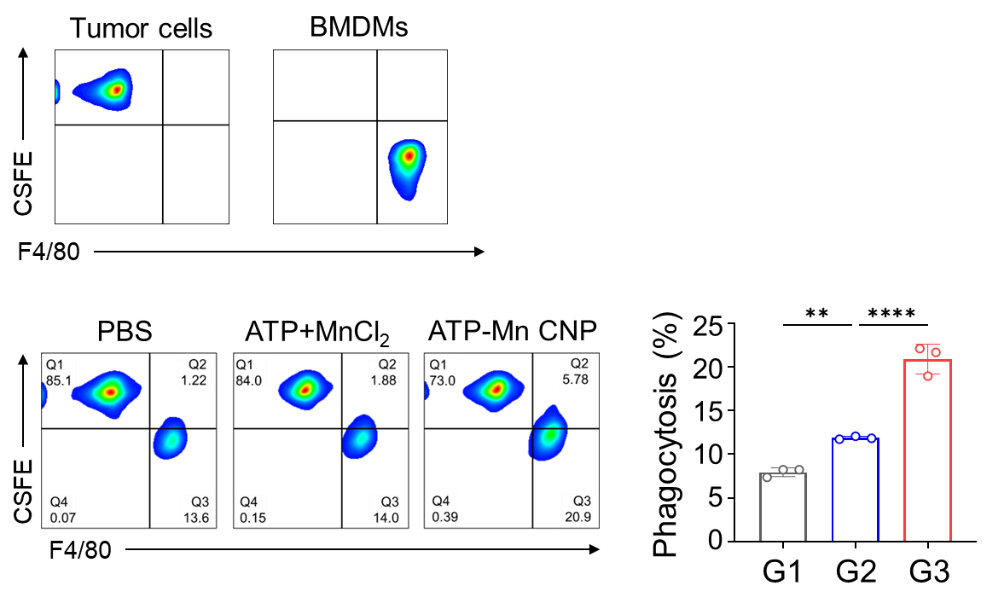


**Figure S17.** Flow cytometry analysis of BMDMs phagocytosis (n = 3).


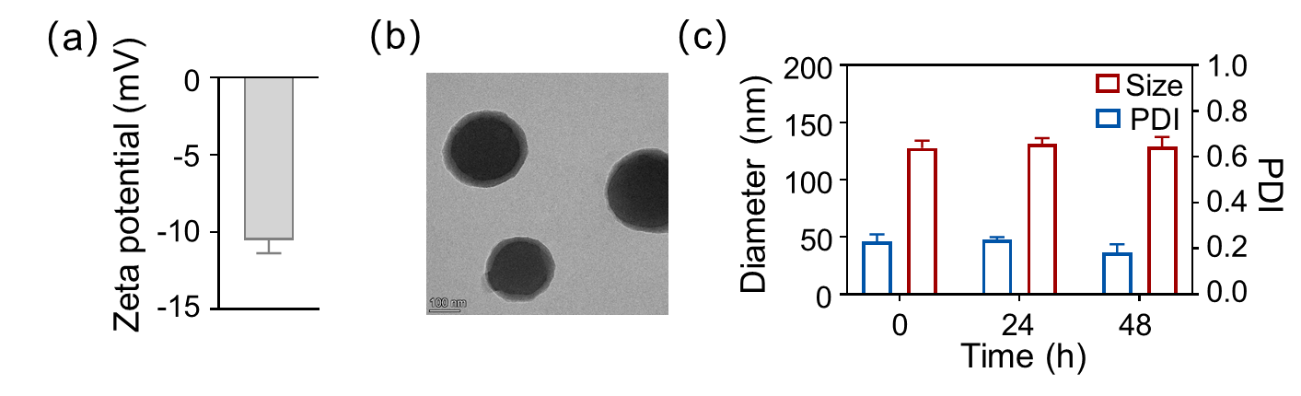


**Figure S18.** (a) Zeta potential of TPP-Mn CNP (n = 3). (b) TEM image of TPP-Mn CNP. Scale bar = 100 nm. (c) Stability evaluation of TPP-Mn CNP in 10% FBS over 2 days (n = 3).


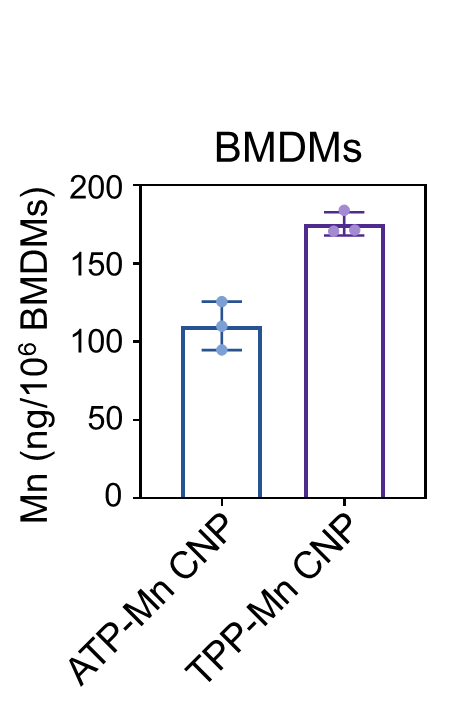


**Figure S19.** BMDMs were incubated with ATP-Mn CNP or TPP-Mn CNP at equivalent Mn concentrations for 24 h. Intracellular manganese levels were quantified by inductively coupled plasma mass spectrometry (ICP-MS) (n = 3).


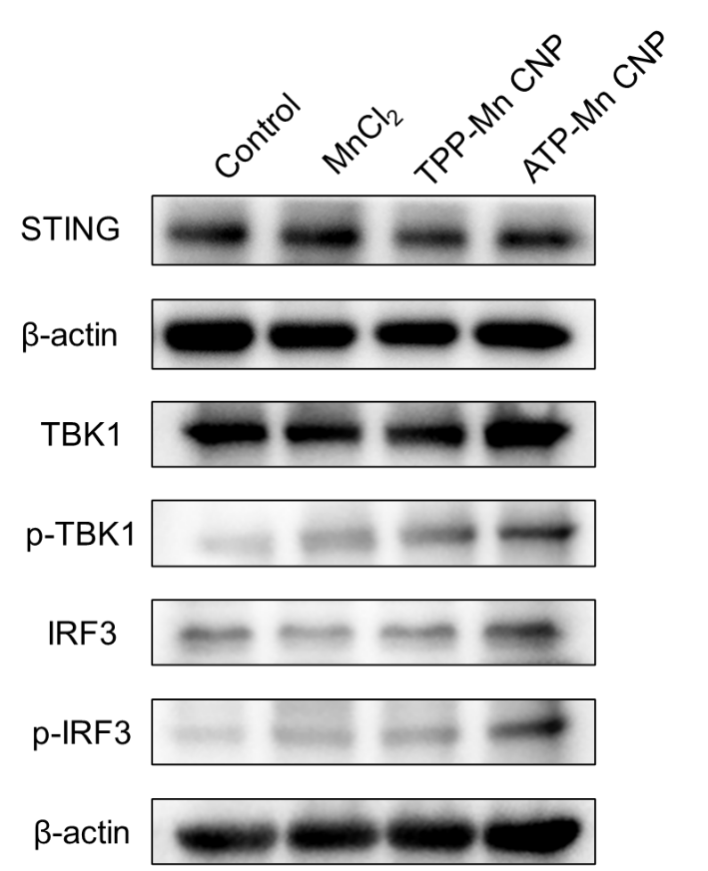


**Figure S20.** Western blot analysis of the expression of STING, TBK1, p-TBK1, IRF3, and p-IRF3 proteins in BMDMs after treatment.


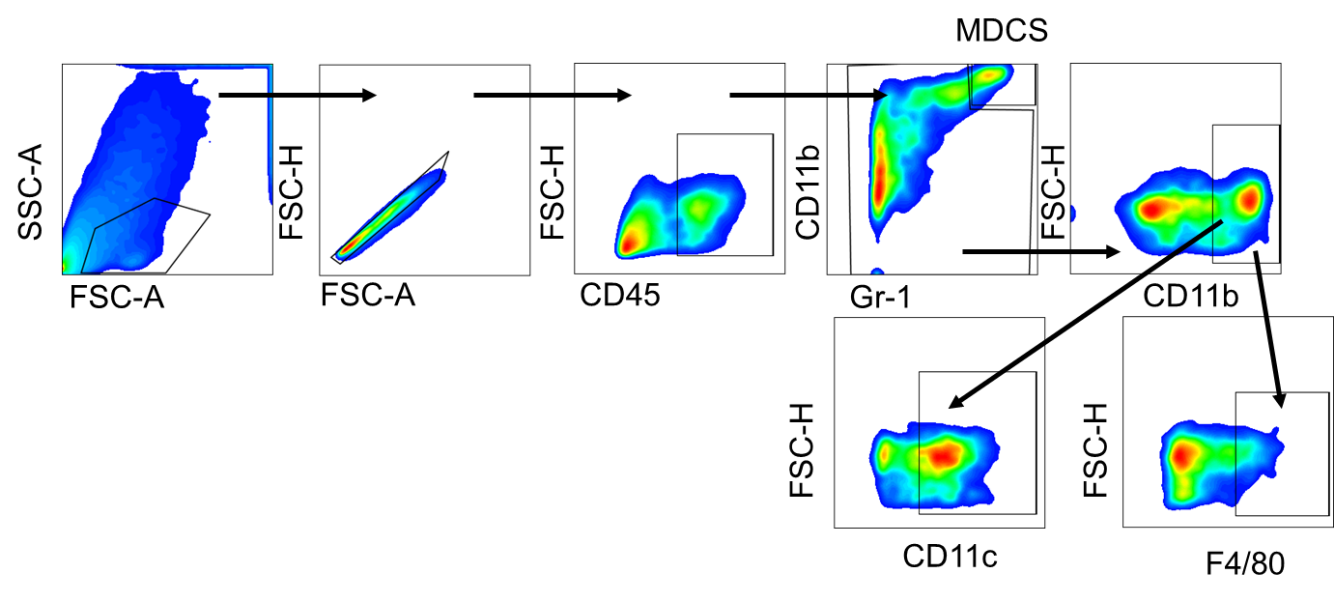


**Figure S21.** Representative flow gate strategies of different immune cells of tumor tissues and tumor-draining lymph nodes after the treatment.

**
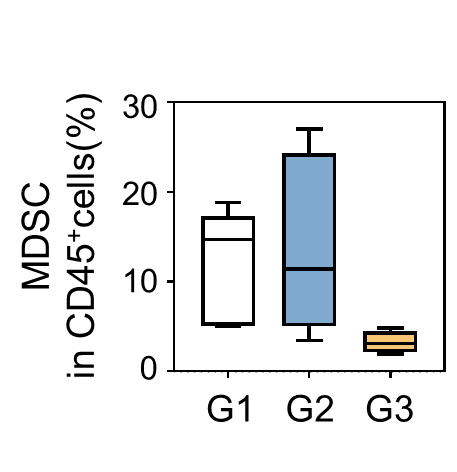
**

**Figure S22.** The ratio of MDSCs in tumors from mice treated with PBS, MnCl_2_ + ATP, and ATP-Mn CNP quantified by flow cytometry (n = 5).

**
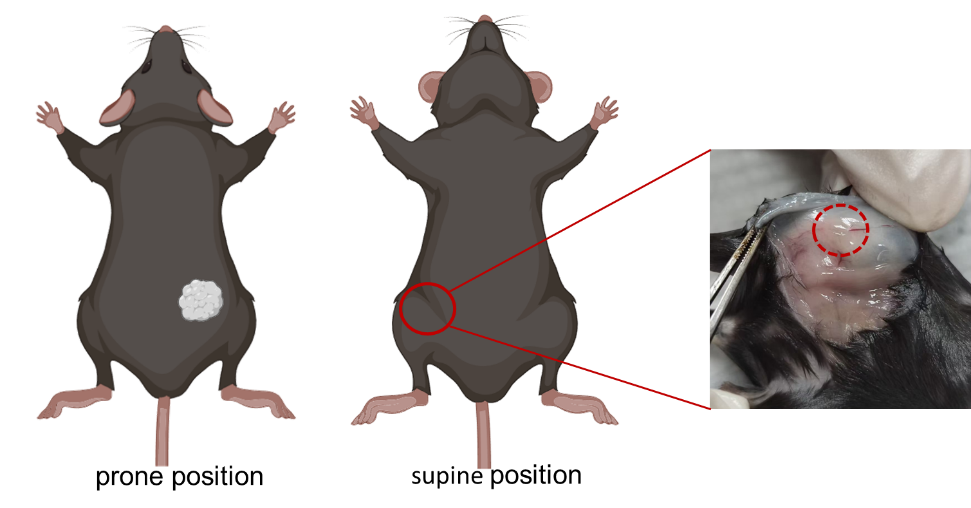
**

**Figure S23.** Schematic diagram of tumor-draining lymph nodes in mice.


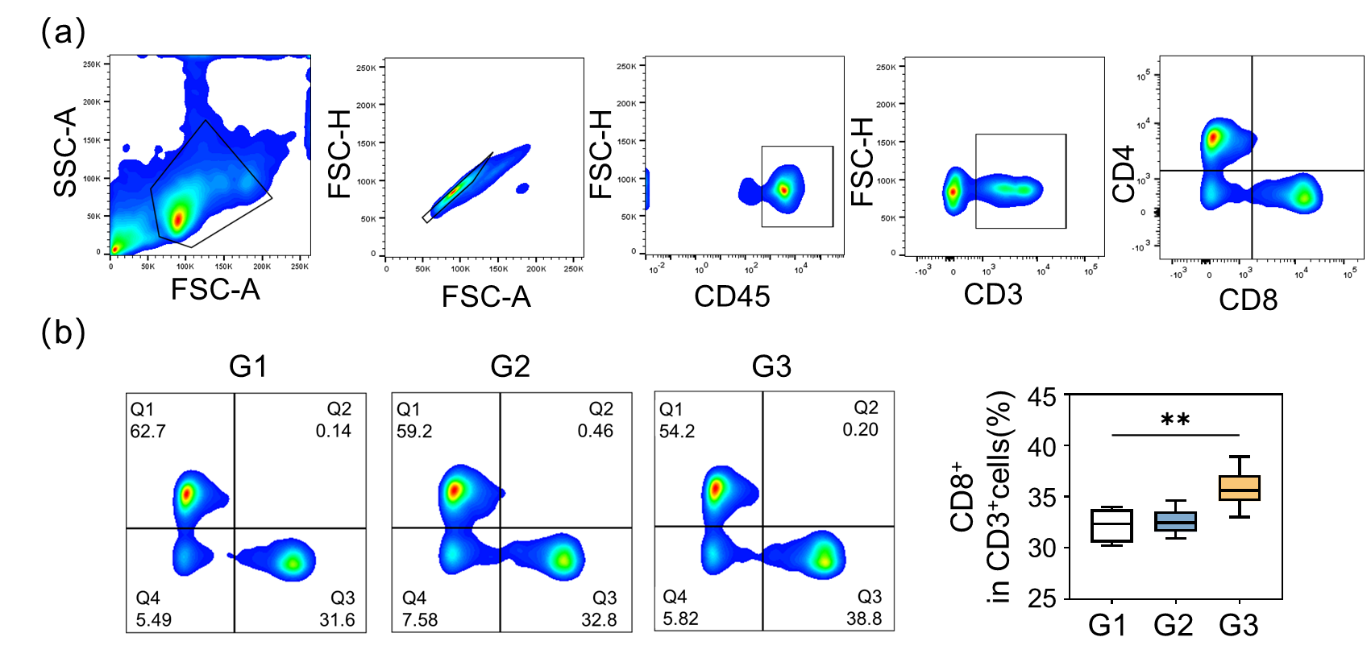


**Figure S24.** (a) Representative flow gate strategies of different immune cells of spleens. (b) The percentage of CD8^+^ T cells in spleens from various treatment groups (n = 5).


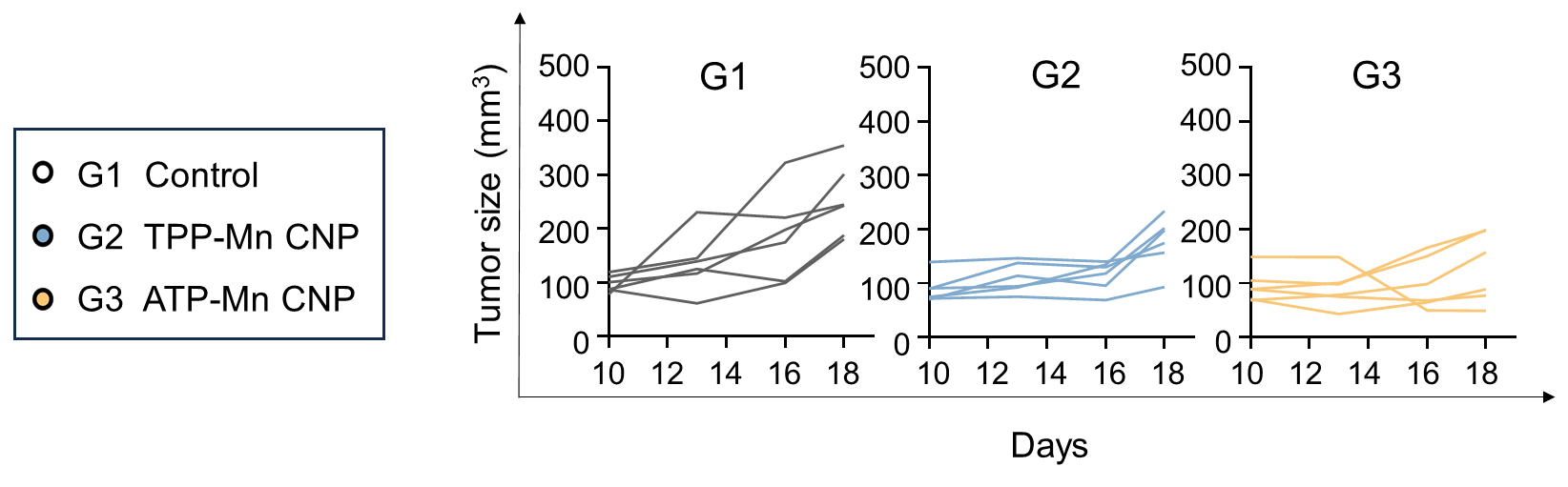


**Figure S25.** Spider plots showing individual tumor growth curves for each mouse across the treatment groups (n = 6).


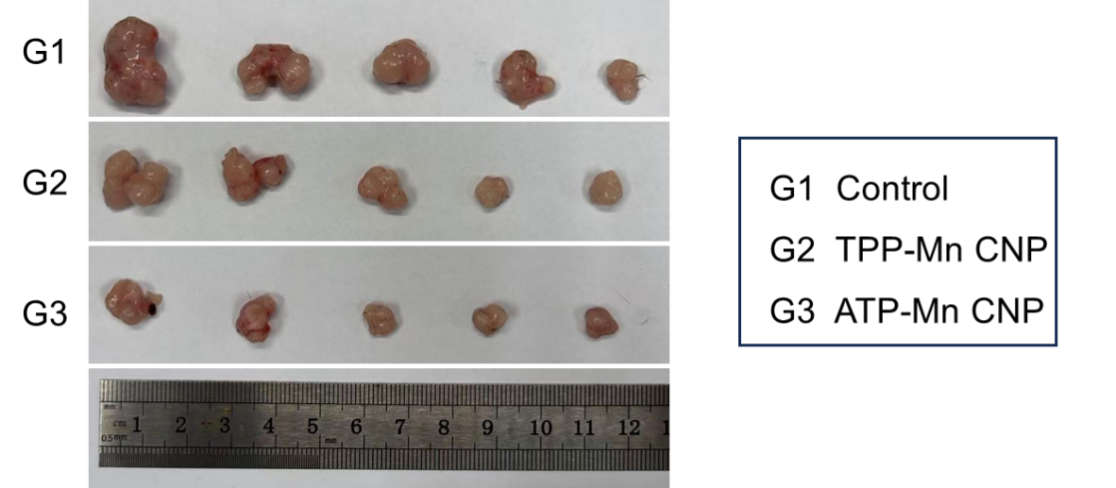


**Figure S26.** Representative tumor images of different treatment groups (n = 5)


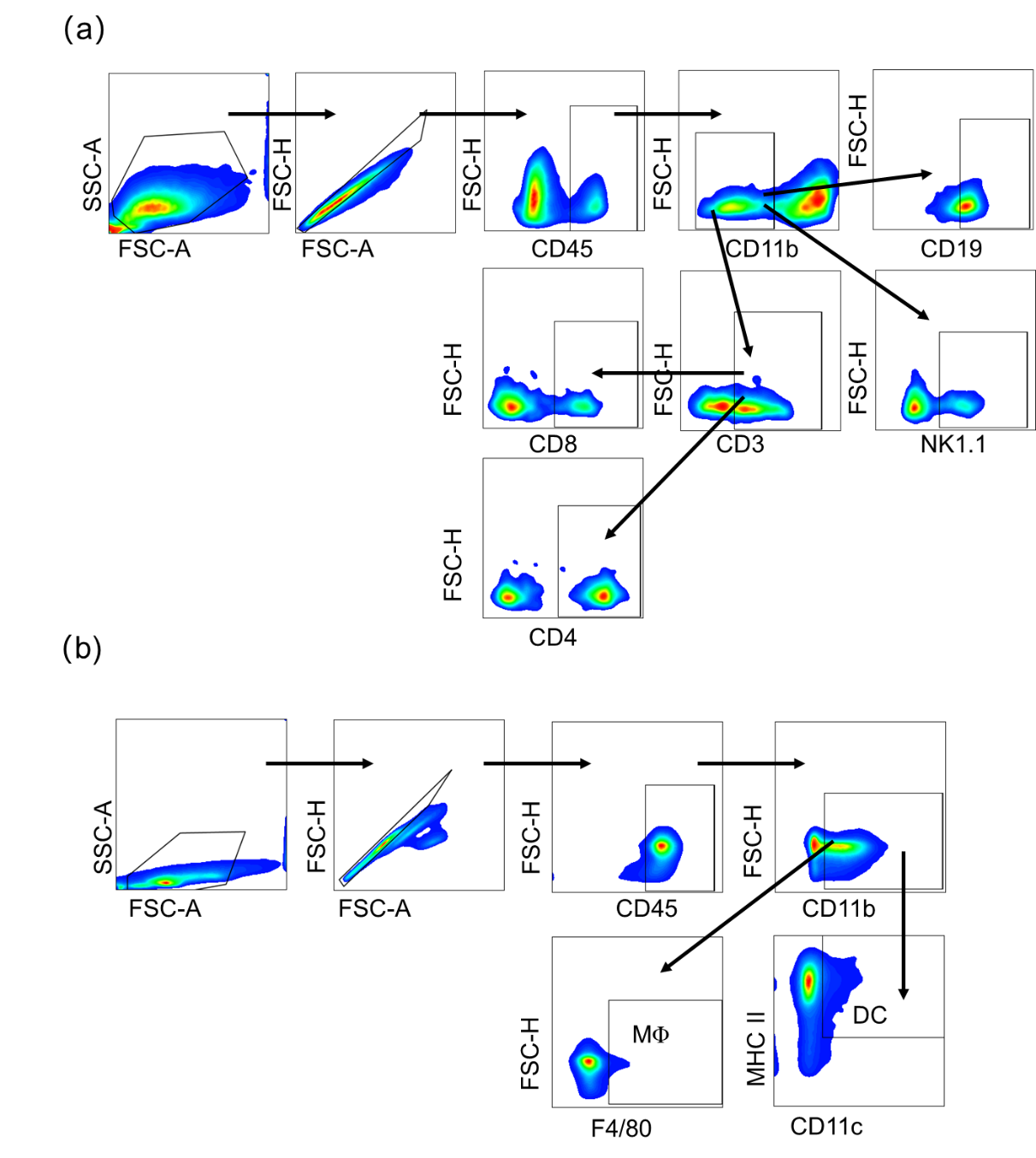


**Figure S27.** (a-b) Representative flow gate strategies of different immune cells of tumor tissues and tumor-draining lymph nodes after the treatment.


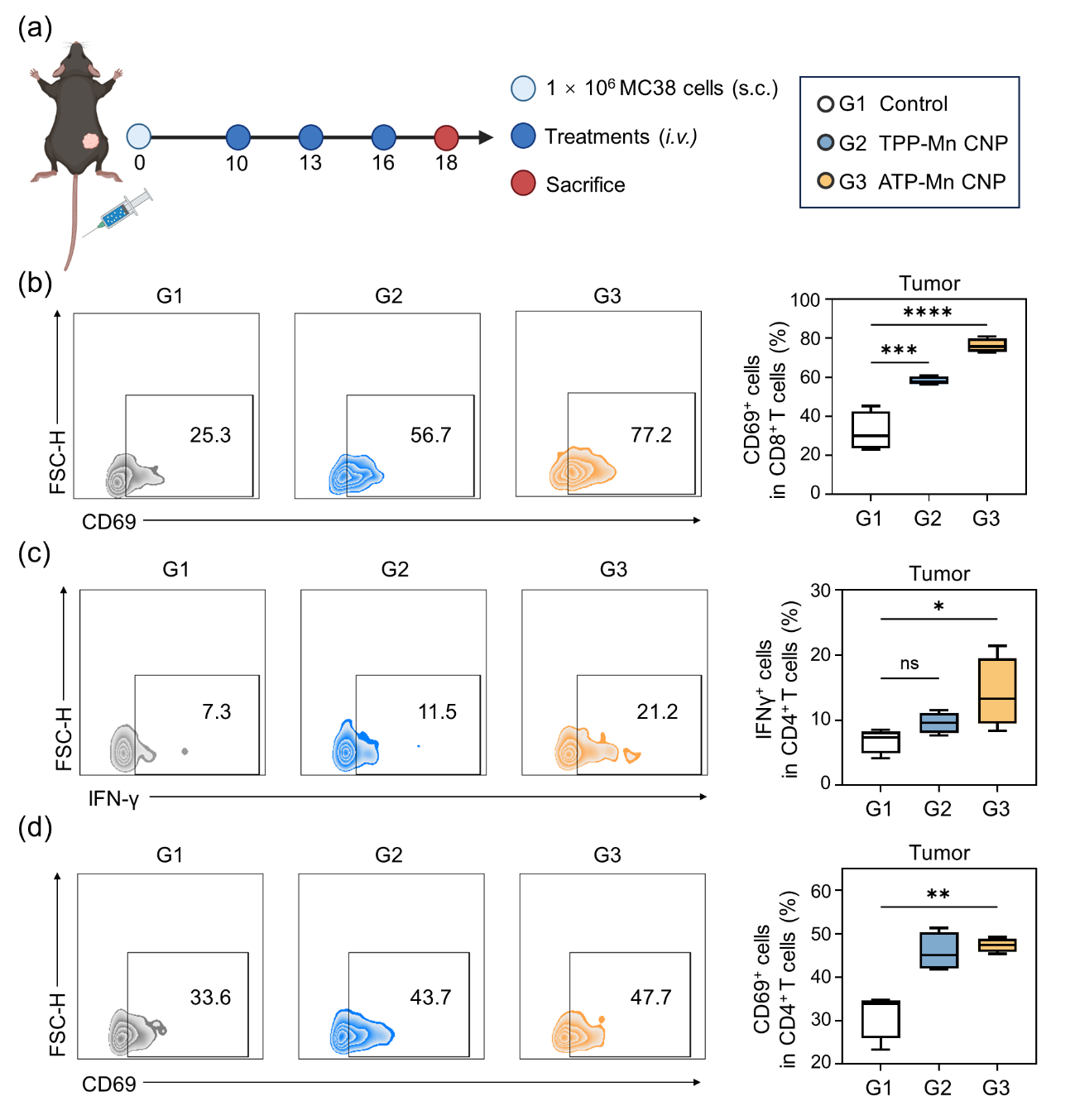


**Figure S28.** (a) Schematic illustration of the treatment process. (b) Representative flow cytometry plots and percentages of CD69^+^ CD8^+^ T cells in tumor immune microenvironment (n = 4). (c) Representative flow cytometry plots and percentages of IFNγ^+^ CD4^+^ T cells in tumor immune microenvironment (n = 4). (d) Representative flow cytometry plots and percentages of CD69^+^ CD4^+^ T cells in tumor immune microenvironment (n = 4).


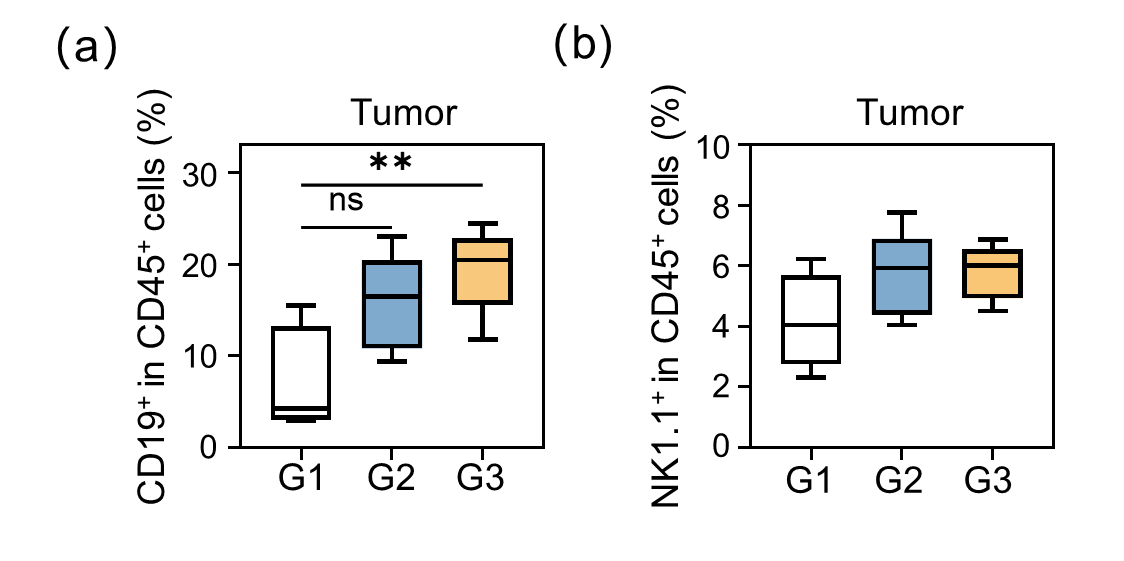


**Figure S29.** (a) Percentages of B cells in tumor immune microenvironment (n = 5). (b) Percentages of NK cells in tumor immune microenvironment (n = 5).


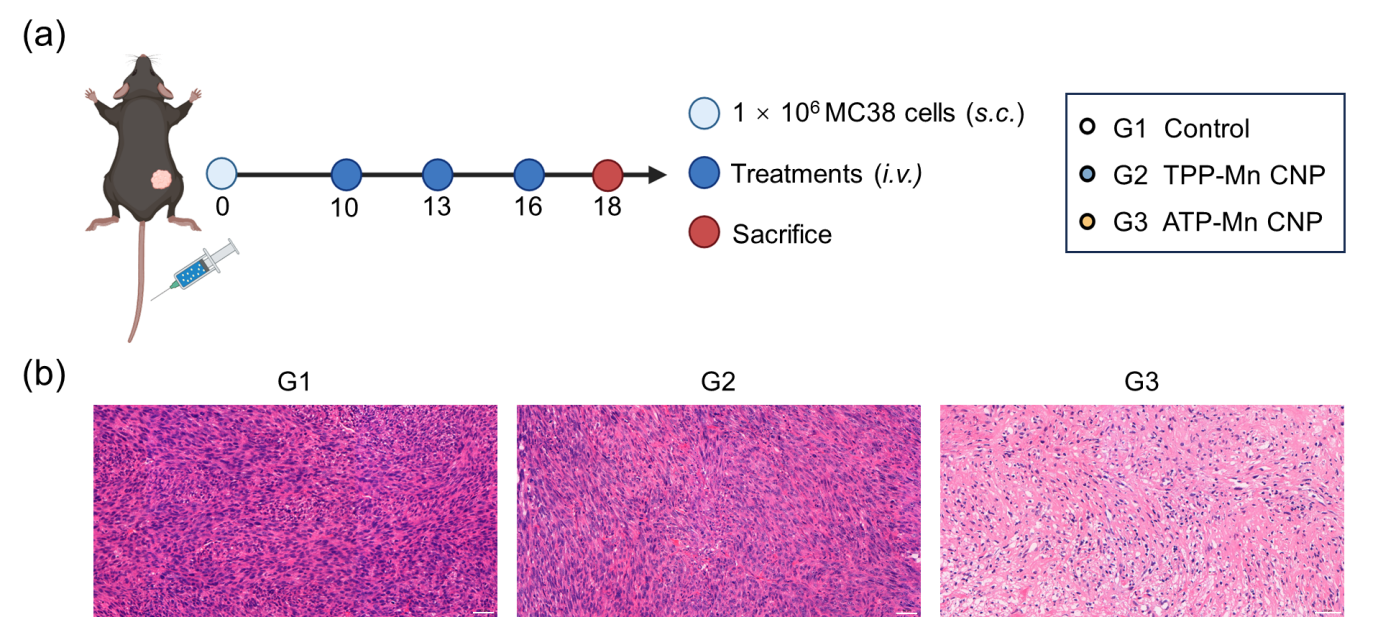


**Figure S30.** (a) Schematic illustration of the treatment process. (b) Representative H&E staining images of tumor from the indicated groups. Scale bars = 50 μm.


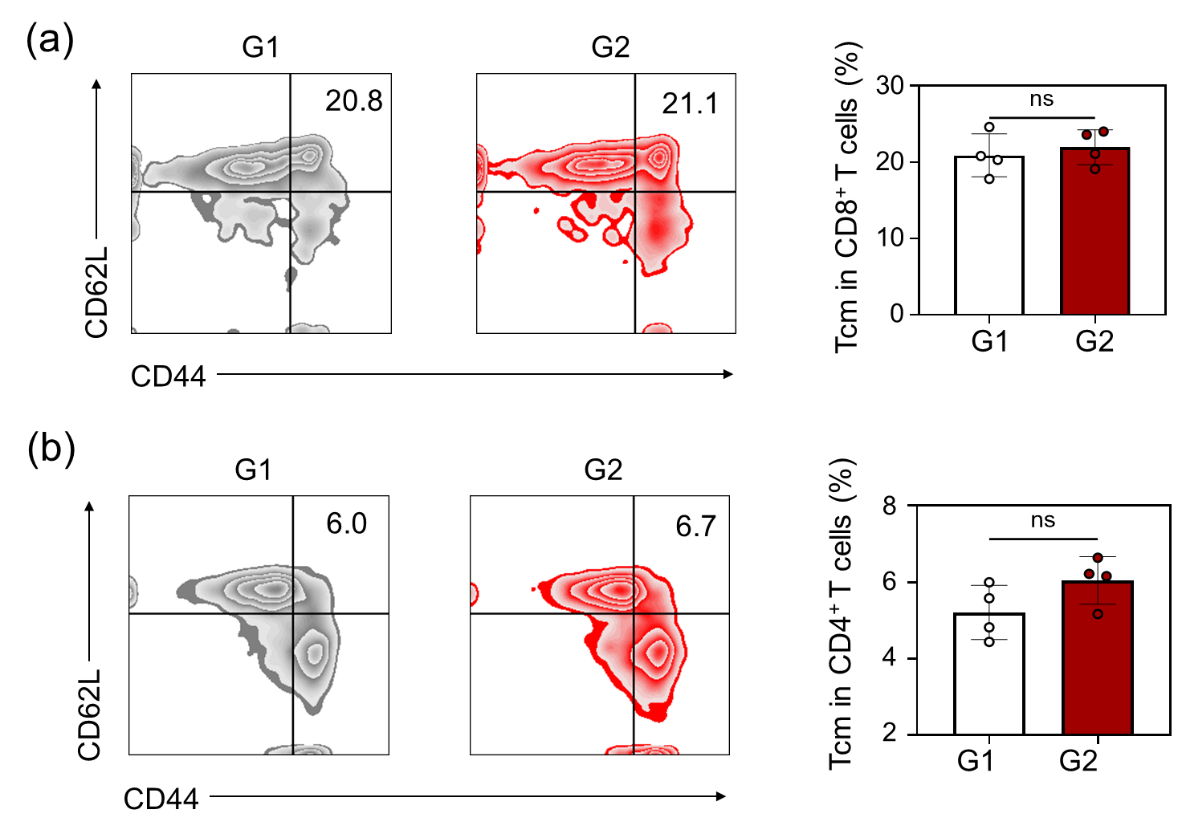


**Figure S31.** (a) Representative flow cytometry plots and percentages of Tcm in CD8^+^ T cells in spleens of different group mice (n = 4). (b) Representative flow cytometry plots and percentages of Tcm in CD4^+^ T cells in spleens of different group mice (n = 4).


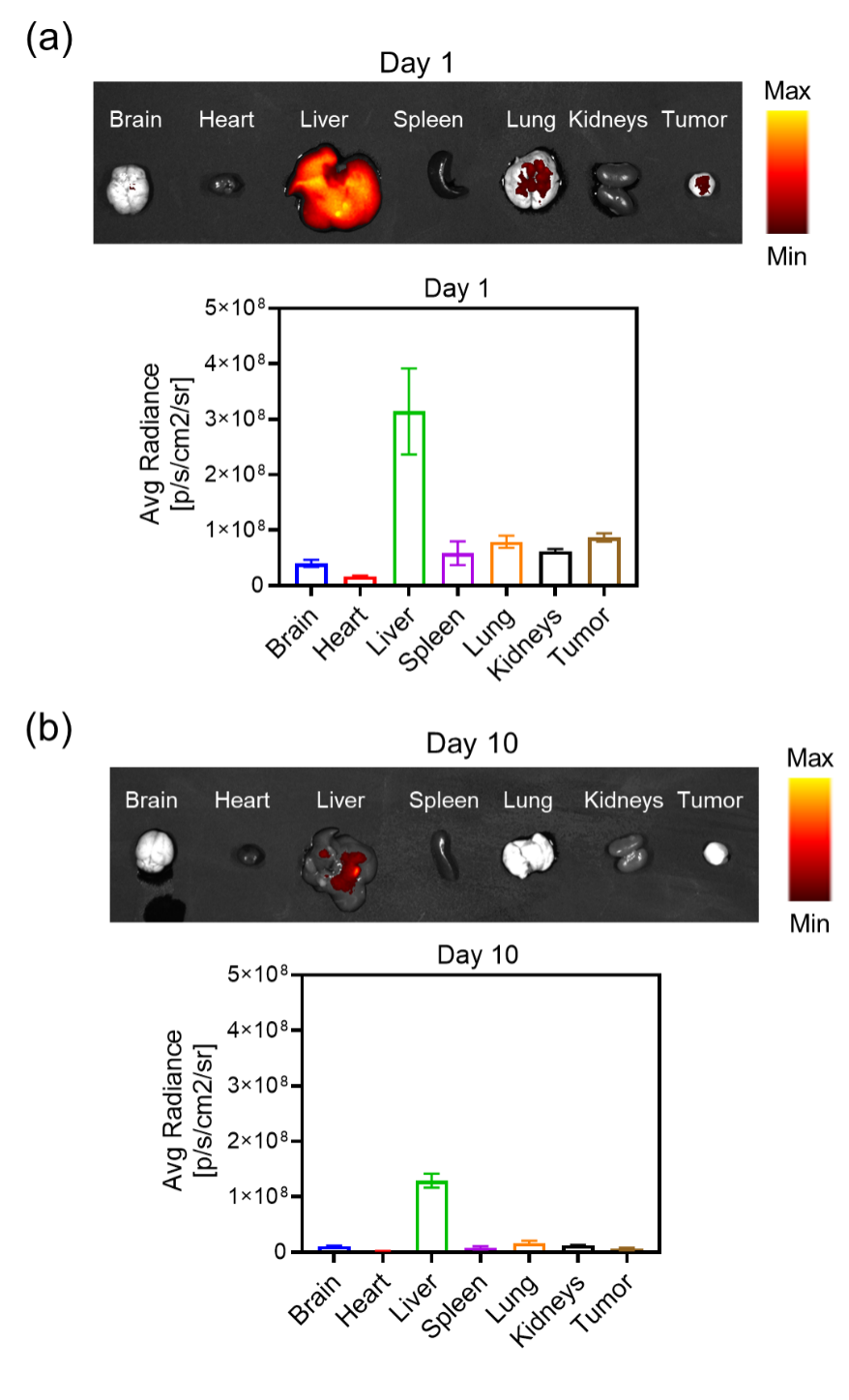


**Figure S32.** (a) *Ex vivo* imaging of major organs 1 day after the injection (n = 3). (b) *Ex vivo* imaging of major organs 10 day after the injection (n = 3).

**Table S1**. Primer sequences used in RT-qPCR analysis

| Primers | Sequences |
| --- | --- |
| *Nos2* | FW: 5’-GAGACAGGGAAGTCTGAAGCAC-3’  RV: 5’-CCAGCAGTAGTTGCTCCTCTTC-3’ |
| *Arg1* | FW: 5’-CTCCAAGCCAAAGTCCTTAGAG-3’  RV: 5’-AGGAGCTGTCATTAGGGACATC-3’ |
| *Cxcl10* | FW: 5’-TCACTCCCCTTTACCCAGTG-3’  RV: 5’-GTCGCACCTCCACATAGCTT-3’ |
| *Ifnb* | FW: 5’-CCCTATGGAGATGACGGAGA-3’  RV: 5’-CTGTCTGCTGGTGGAGTTCA-3’ |
| *Il1b* | FW: 5’-TGTAATGAAAGACGGCACACC-3’ RV: 5’-TCTTCTTTGGGTATTGCTTGG-3’ |
| *Cd206* | FW: 5’-CTCTGTTCAGCTATTGGACGC-3’ RV: 5’-CGGAATTTCTGGGATTCAGCTTC-3’ |
| *Isg15* | FW: 5’-AAGCAGCCAGAAGCAGACTC-3’ RV: 5’-TAAGACCGTCCTGGAGCACT-3’ |
| *Isg20* | FW: 5’-GCCGAGAAGTGGAAACAGAG-3’  RV: 5’-CTCGGGTCGGATGTACTTGT-3’ |
